# From Charge Storage Rulebook Rewriting to Commercial Viability of Zinc‐Manganese Batteries

**DOI:** 10.1002/advs.202509520

**Published:** 2025-07-02

**Authors:** Xinhua Zheng, Bibo Han, Shikai Liu, Shiya Huang, Song Wu, Mingyan Chuai, Faxing Wang, Yuping Wu

**Affiliations:** ^1^ School of Materials Science and Engineering Henan University of Technology Zhengzhou Henan 450001 China; ^2^ State Key Laboratory of Structural Chemistry Fujian Institute of Research on the Structure of Matter Chinese Academy of Sciences Fuzhou Fujian 350002 China; ^3^ Confucius Energy Storage Lab School of Energy and Environment & Z Energy Storage Center Southeast University Nanjing Jiangsu 210096 China

**Keywords:** energy storage mechanism, large‐scale energy storage, pouch cell, zinc‐manganese oxide batteries

## Abstract

Aqueous zinc‐manganese oxide (Zn‐MNO) batteries represent a compelling solution for grid‐scale energy storage due to their inherent safety, cost‐effectiveness and ecological compatibility. However, the commercialization of this technology faces critical challenges including insufficient electrode durability, limited areal capacity output, and fundamentally ambiguous charge storage principles, which collectively hinder practical implementation. Through systematic mechanistic investigation, a previously overlooked phase evolution paradigm is revealed. It involves that Mn_3_O_4_ cathode undergoes a partially in situ phase transition to MnO_2_ during the initial charging process, forming a hybrid Mn_3_O_4_/MnO_2_ cathode. This self‐optimized heterostructure synergistically combines structural reinforcement frameworks with enhanced ion‐transport networks, enabling exceptional cycling stability over 4,500 cycles while maintaining record‐high areal capacity (10 mAh cm^−2^). The clarified dual‐ion (H^+^/Zn^2+^) coordination mechanism and stabilized Mn^2+^/MnO_2_ redox chemistry establish new design principles for manganese‐based cathodes. More importantly, unprecedented scalability is demonstrated through constructing pouch cells (200 mAh) with 1000‐cycle durability, achieving a practical energy density of 54 Wh kg^−1^. The integrated solar‐powered battery system exhibits remarkable operational safety under extreme conditions (piercing, cutting), representing one of the most practically viable Zn‐MNO batteries reported to date. This work bridges fundamental mechanistic understanding with industrial‐grade device engineering, charting a concrete pathway toward terawatt‐hour scale renewable energy storage.

## Graphical abstract

The Mn_3_O_4_ cathode undergoes a partially in situ phase transition to MnO_2_ during the initial charging process, forming a hybrid Mn_3_O_4_/MnO_2_ cathode. The Mn_3_O_4_ provides a robust supporting structure for MnO_2_ to enhance the cathode stability upon H^+^/Zn^2+^ co‐insertion/extraction. Meanwhile, Mn_3_O_4_ provides additional active sites for Mn^2+^/MnO_2_ chemistry, greatly boosting the battery areal capacity.

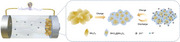

## 1 Introduction

The development of zinc‐manganese oxide (Zn‐MNO) batteries has been going on for over a century, and their primary battery systems in alkaline electrolytes were widely applied in consumer products.^[^ ^1^ ^]^ Since 2012, Kang and coworkers^[^ ^2^ ^]^ proposed a reversible insertion/extraction of Zn^2+^ in α‐MnO_2_ in mildly acidic aqueous electrolytes. Since then, the research on rechargeable Zn‐MNO batteries for energy storage applications has attracted significant interest, expecting a preferred electrochemical energy storage device in the post‐lithium era.^[^ ^3^ ^]^ To match the high specific capacity (820 mAh g^−1^, 5855 mAh cm^−3^) and reversibility of Zn anodes, it is crucial to develop energetic MNO cathodes to promote the practical applications of Zn‐MNO batteries.^[^ ^4^ ^]^

The advanced nature of MNO can be attributed to the multiple valence states of Mn (including Mn^2+^, Mn^3+^, Mn^4+^, Mn^5+^, and Mn^7+^)^[^ ^5^, ^6^ ^]^ and its abundance in MNO materials (MnO, MnOOH, MnO_2_, Mn_2_O_3_, Mn_3_O_4_, and their derivatives).^[^ ^6^ ^]^ Amongst, MnO_2_ cathodes were commonly investigated in Zn‐MNO batteries, mainly including α‐MnO_2_ (belonging to the body‐centered tetragonal crystal system) with a tunneling structure (2 × 2, ∼0.46 nm × 0.46 nm), and δ‐MnO_2_ (belonging to the monoclinic crystal system) with a 2D layered structure (with a layer spacing of ∼0.7 nm).^[^ ^7^, ^8^ ^]^ The generally accepted energy storage mechanism of the MnO_2_ cathode was Zn^2+^ and H^+^ co‐insertion/extraction, and some reports suggested that there is a H^+^‐ dominated intercalation reaction.^[^ ^9^, ^10^ ^]^ The specific capacities and reversibility of the batteries depend critically on multiple factors including the structural spatial distribution, stability, charge transfer pathways, phase composition, and microstructural features of the MnO_2_ cathode.^[^ ^11^, ^12^, ^13^, ^14^ ^]^ Interestingly, the recently achieved initial specific capacity of the MnO_2_ cathode is approaching its theoretical value of 308 mAh g^−1^ at small current densities (< 0.2 A g^−1^), while the cycling stability over thousands of cycles at higher current densities (> 2 A g^−1^).^[^ ^15^, ^16^ ^]^ For instance, Li and coworkers^[^ ^17^ ^]^ regulated hydrogen bonds of MnO_2_ cathode by pre‐intercalation of NH_4_^+^ for Zn ion storage, which achieved a large specific capacity of 287.9 mAh g^−1^ at 0.1 A g^−1^ and ultra‐high rate performance (99.4 mAh g^−1^ at 6.0 A g^−1^). Their excellent electrochemical performance is generally harvested at low mass loading, high current densities, and severe capacity degradation. This can be ascribed to the disadvantages of MnO_2_ cathode, including irreversible phase transitions, volume expansion, electrode structural collapse, and dissolution.^[^ ^18^, ^19^ ^]^ Therefore, it is necessary to explore alternative MNO cathodes for energetic Zn‐MNO batteries.

Recently, researchers have made much progress in developing novel MNO cathodes and using their unique structures and energy storage mechanisms to achieve advanced Zn‐MNO batteries.^[^ ^20^, ^21^ ^]^ For instance, Xiao and coworkers^[^ ^22^ ^]^ designed a MnO cathode for reversible Zn^2+^ insertion/extraction, and the constructed Zn‐MnO battery achieved 1000 stable cycles. Zhang and coworkers^[^ ^14^ ^]^ reported an amorphous MnO_2_ nanosheet with abundant oxygen vacancies. The assembled Zn‐MnO_2_ battery delivered a specific capacity of 324 mAh g^−1^ at 0.5 A g^−1^ and achieved a capacity retention rate of 86.2% after 1000 cycles. Long and coworkers^[^ ^23^ ^]^ developed a MnOOH cathode for Zn^2+^/H^+^ co‐insertion/extraction, and the built Zn‐MnOOH battery achieved a stable 500 cycles with a specific capacity of 108 mAh g^−1^ at 0.5 A g^−1^. Lu and coworkers^[^ ^24^ ^]^ used Mn_2_O_3_ as a cathode to assemble a rechargeable Zn‐Mn_2_O_3_ battery, which exhibited good cycling stability with 91% capacity retention at 1 A g^−1^ after 1000 cycles. In contrast to MnO_2_, MnO, and Mn_2_O_3_, the spinel crystal framework of Mn_3_O_4_ offers inherent resistance to phase collapse through tetrahedral‐octahedral cation coordination, and also its mixed Mn^2+^/Mn^3+^ valence enables multi‐electron transfer reactions.^[^ ^25^, ^26^ ^]^ Xu and coworkers^[^ ^27^ ^]^ designed a nitrogen‐doped Mn_3_O_4_, which elongated the Mn─O bond in the crystal and suppressed the lattice J‐T aberration induced by [MnO_6_] octahedra. The constructed Zn‐Mn_3_O_4_ battery exhibited a high specific capacity of 420 mAh g^−1^ at a current density of 1 A g^−1^, and demonstrated a stable 1000 cycles with a capacity retention of 90.34%. Although the research on these novel Zn‐MNO batteries has achieved much progress, their areal capacities, cycling stability, energy storage mechanism, and the amplification of battery capacity have not been well validated.^[^ ^28^, ^29^ ^]^ The current challenges of MNO cathodes can be summarized as poor electrical conductivity suppressing reaction kinetics and irreversible phase transitions/volume expansion/structural collapse/Mn dissolution, eventually leading to bad rate capability and rapid capacity decay.^[^ ^30^ ^]^ Moreover, the achieved limiting areal capacities (generally less than 1 mAh cm^−2^) hinder the development of Zn‐MNO in practical applications.

Herein, we develop a high‐performance Zn‐Mn_3_O_4_ battery by optimizing the synthesis of a Mn_3_O_4_ cathode with an ultrahigh areal capacity (> 10 mAh cm^−2^) and revisiting its energy storage mechanism. Theoretical calculations and characterizations confirm that Mn_3_O_4_ undergoes a partial phase transition to MnO_2_ during the initial charging process and forms a hybrid Mn_3_O_4_/MnO_2_ cathode. The excellent structure stability and high reversibility for Zn^2+^/H^+^ insertion/extraction suggest that the hybrid cathode, after the initial phase transition, inherits the advantages of MnO_2_ and Mn_3_O_4_. Moreover, the cathode offers additional active areas for Mn^2+^/MnO_2_ chemistry, effectively expanding the areal capacity of the battery. As a result, the Zn‐Mn_3_O_4_ battery demonstrates exceptional cycling stability, maintaining high reversibility of over 4500 cycles at 1 A g^−1^ and sustaining stable operation at a high areal capacity of 10 mAh cm^−2^. Moreover, the enlarged Zn‐Mn_3_O_4_ pouch cell achieves 1000 stable cycles, while successfully integrating with solar energy and showcasing excellent safety. The proposed Mn_3_O_4_ cathode with novel chemistry is expected to propel the development of Zn‐MNO rechargeable batteries and contribute to large‐scale energy storage applications.

## 2 Results and Discussion

### 2.1 Designing of the Mn_3_O_4_ Cathode

Mn_3_O_4_ is a spinel structure and belongs to a cubic crystal system, while its structural formula can be written as Zn^2+^(Mn^3+^)_2_O_4_.^[^ ^31^ ^]^ The oxygen ions are in the cubic closest packing, and the divalent and trivalent manganese ions occupy the tetrahedral and octahedral interstitials, respectively.^[^ ^32^ ^]^ The gap in this structure is more likely to provide suitable channels and locations for ion insertion. Furthermore, the ion arrangement in Mn_3_O_4_ is compact and regular, resulting in a relatively high lattice density in the crystal.^[^ ^33^ ^]^ Moreover, the combination of chemical bonds formed by different valence states of Mn─O bonds in Mn_3_O_4_ makes it more resistant to the chemical bond breaking and structural variation upon chemical environment changes.^[^ ^25^ ^]^ The chemical bonds in Mn_3_O_4_ consisting of Mn^2+^, Mn^3+^, and O are flexible, and active ions (Zn^2+^ and H^+^) in the electrolyte can easily interact with the lattice around Mn^2+^ and Mn^3+^, resulting in the preferable of ion storage.^[^ ^34^ ^]^ In addition, the 3D ion diffusion channels provided by the spinel structure of Mn_3_O_4_ may effectively buffer the volume changes during embedding and detachment, contributing to the cycling stability of cathode. Therefore, the optimized design of the Mn_3_O_4_ cathode is expected to achieve practically energetic Zn batteries.^[^ ^26^, ^31^, ^35^ ^]^

**Figure 1:**
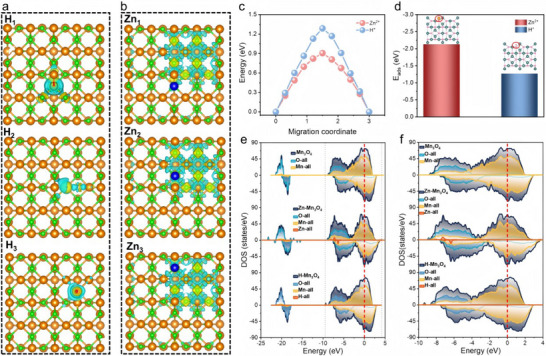
Theoretical calculations of the energy storage mechanisms of Mn_3_O_4_. Differential charge densities of a) H^+^ and b) Zn^2+^ insertion into Mn_3_O_4_. c) Migration energy barriers for Zn^2+^ and H^+^ in Mn_3_O_4_. d) Adsorption energy of Zn^2+^ and H^+^ on Mn_3_O_4_. Density of states (DOS) of Mn_3_O_4_, Zn‐Mn_3_O_4,_ and H‐Mn_3_O_4_ in e) Overall energy, and f) localized magnification of DOS near the Femi energy level.

To clarify the energy storage mechanism of Mn_3_O_4_ cathode for Zn‐MNO batteries, theoretical calculations based on the density functional theory (DFT) were preliminarily carried out. Figure S1 (Supporting Information) shows the geometrical models of Mn_3_O_4_ at different views, where brown is elemental Mn and green is elemental O. We first evaluated the potential of Mn_3_O_4_ for H^+^ and Zn^2+^ storage. As shown in Figure S2 (Supporting Information), both H^+^ and Zn^2+^ can be successfully stored in Mn_3_O_4_, and the transport path of Zn^2+^ is shorter than H^+^. The differential charge densities diagrams show the distribution of electrons gained and lost by Zn^2+^ and H^+^ in three different states (**Figure** **1**a,b). The results confirm that Zn^2+^ has a wider range of charge influence in Mn_3_O_4_, which suggests that Zn^2+^ is more easily transported in Mn_3_O_4_ and thus contributes to the capacity by being more favorable for energy storage. Further evidence is provided by the migration pathways of Zn^2+^ and its corresponding energy value. The energy barrier for Zn^2+^ migration in the Mn_3_O_4_ cathode is lower than that of H^+^, suggesting that Zn^2+^ ions migrate more readily and thus contribute primary capacity to the battery (Figure 1c). Figure 1d displays that the adsorption energy of Zn^2+^ in Mn_3_O_4_ is −2.2 eV, which is significantly lower than that of H^+^ (−1.3 eV). It suggests that Zn^2+^ is more easily adsorbed than H^+^ in Mn_3_O_4_ cathode. Dirac point statistics exhibit the Mn_3_O_4_, Zn‐Mn_3_O_4_, and H‐Mn_3_O_4_ are 8, 26, and 15, respectively (Figure S3, Supporting Information). It suggests that the Zn^2+^ and H^+^ insertion into the Mn_3_O_4_ can effectively enhance their stability and conductivity. The density of states (DOS) of Mn_3_O_4_, Zn‐Mn_3_O_4,_ and H‐Mn_3_O_4_ are displayed in Figure 1e,f, where Figure 1e is the DOS in the overall energy, and Figure 1f is the localized magnification of DOS near the Femi energy level. Zn‐Mn_3_O_4_ and H‐Mn_3_O_4_ have wider energy distribution regions compared to Mn_3_O_4_. This implies that the insertion of Zn^2+^ and H^+^ leads to an increase in the charge‐leap energy levels, which in turn leads to an increase in the charge storage space. Moreover, after the Zn^2+^ and H^+^ insertion into the Mn_3_O_4_, the peaks of the Zn‐Mn_3_O_4_ and H‐Mn_3_O_4_ near the Femi energy level become sharp, especially after Zn^2+^ insertion (Figure 1f). This indicates that the ionic inserting enhances the off‐domain nature of the material, thus increasing the electrochemical activity. Overall, the theoretical calculations reveal it is expected to realize a highly reversible carrier for Zn^2+^/H^+^ energy storage in Mn_3_O_4_ for Zn‐MNO battery. Moreover, the insertion of Zn^2+^ seems to occur more readily compared to H^+^, and thus the realized reversible capacity is more likely to be dominated by Zn^2+^.

### 2.2 Working Mechanism of the Zn‐Mn_3_O_4_ Battery

**Figure 2:**
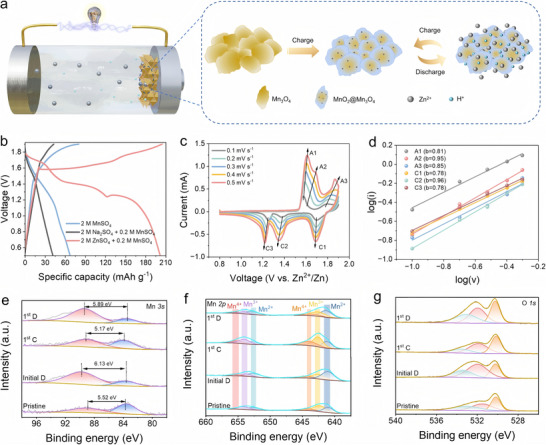
Energy storage mechanism of Mn_3_O_4_. a) Schematic diagram of the energy storage mechanism of the Zn‐Mn_3_O_4_ battery. b) Charge/discharge curves of Zn‐Mn_3_O_4_ batteries in different electrolytes. c) CV curves of the Zn‐Mn_3_O_4_ batteries at different scan rates. d) A linear plot and fitting of log (*v*) versus log (*i*). XPS spectra of Mn_3_O_4_ at e) Mn *3s*, f) Mn *2p*, and g) O *1s* under different charging and discharging states, where “C” and “D” represent charge and discharge states, respectively.

Next, experimental characterizations were used to further understand the energy storage mechanism of the Mn_3_O_4_ cathode. Instead of the previously reported Mn_3_O_4_, our results prefer to consider the reaction mechanism as shown in **Figure** **2**a. The Zn‐Mn_3_O_4_ battery was constructed via a Mn_3_O_4_ coated carbon felt, a Zn foil anode, and a low‐cost hybrid electrolyte of 2 M ZnSO_4_ and 0.2 M MnSO_4_. With the gradual increase in voltage during charging, the stability window of Mn_3_O_4_ may be breached, leading to its partially oxidized transformation to MnO_2_.^[^ ^36^ ^]^ Another reason is that, during charging with the ions extraction from Mn_3_O_4_, Mn loses electrons resulting in charge rearrangement, and meanwhile, to adapt to the changes in ionic radius and charge state, Mn_3_O_4_ is transformed to MnO_2_.^[^ ^37^, ^38^ ^]^ As a result, the Mn_3_O_4_ cathode is partially converted to MnO_2_ during initial charging, constructing a hybrid Mn_3_O_4_/MnO_2_ cathode. During the discharge process, Zn^2+^ and H^+^ are inserted into the MnO_2_/Mn_3_O_4_ cathode and lower its valence. Interestingly, the Mn_3_O_4_/MnO_2_ cathode is likely to provide an active area for the deposition of Mn^2+^ to MnO_2_, thus facilitating the Mn^2+^/MnO_2_ reaction and providing additional capacity. In subsequent cycles, this hybrid Mn_3_O_4_/MnO_2_ material always remained in the cathode and continues to perform highly reversible redox chemistry.

Sufficient evidence for the energy storage mechanism of the Mn_3_O_4_ cathode will be provided below. The discharge curves of the Zn‐Mn_3_O_4_ batteries in different electrolytes confirm the specific reaction of the Mn_3_O_4_ cathode (Figure 2b). When discharging the battery in 2 M MnSO_4_, 2 M Na_2_SO_4,_ and 0.2 M MnSO_4_ electrolyte, the battery exhibits the lower discharge specific capacities below 70 mAh g^−1^. When discharging the battery in hybrid 2 M ZnSO_4_ and 0.2 M MnSO_4_ electrolytes, the Zn‐Mn_3_O_4_ batteries exhibit a discharge capacity of over 200 mAh g^−1^. Clearly, the battery shows three distinct platforms, which can be ascribed to Zn^2+^/H^+^ co‐insertion/extraction and Mn^2+^/MnO_2_ chemistries. The higher specific capacity of the lower platform in the presence of Zn^2+^ suggests that the inserted‐type reactions are dominated by Zn^2+^. Moreover, the Mn_3_O_4_/MnO_2_ cathode provides an active area for the deposition of Mn^2+^ to MnO_2_, thus facilitating the Mn^2+^/MnO_2_ reaction and providing additional capacity. Moreover, cyclic voltammetry (CV) measurements at different sweep rates were performed to investigate the electrochemical kinetics performance of the Mn_3_O_4_/MnO_2_ cathode (Figure 2c). Three pairs of redox peaks slightly shifting with the increasing scan rates are attributed to polarization increase during Zn^2+^/H^+^ insertion/extraction (1.2–1.7 V) and Mn^2+^/MnO_2_ (1.7–1.9 V) chemistries. The equation *i* = *av^b^* is used to deliver the relationship between peak current (*i*) and scan rate (*v*), where the values of a and b are two empirical constants. With the increasing sweep rates from 0.1 to 0.5 mV s^−1^, the *b* values of peak A1, A2, A3, C1, C2, C3 of the cathode are 0.81, 0.95, 0.85, 0.78, 0.96, and 0.78, respectively (Figure 2d). It has been extensively demonstrated that when the parameter *b* equals 1, it signifies a surface capacitive‐controlled reaction, whereas *b* = 0.5 is indicative of a diffusion‐controlled reaction.^[^ ^39^, ^40^, ^41^ ^]^ It can be concluded that the ion storage reactions of the Mn_3_O_4_ cathode are ascribed to the combination of both diffusion and capacitive behaviors. It is well established that the peak current (*i*) is composed of capacitive (*k_1_v*) and diffusion‐controlled (*k_2_v^1/2^*) components, which can be described by the equation *i* = *k_1_v* + *k_2_v^1/2^*. Notably, the capacitive contribution increases from 64.52% at a scan rate of 0.1 mV s^−1^ to 77.33% at 0.5 mV s^−1^, indicating the excellent reaction kinetics of the Zn‐Mn_3_O_4_ battery (Figure S4, Supporting Information). The corresponding dQ/dV versus voltage curve also shows three pairs of distinct redox peaks corresponding to the three reactions mentioned above (Figure S5, Supporting Information).

Further evidence is illustrated in X‐ray photoelectron spectra (XPS) and synchrotron radiation measurements. The evolution of structure and valence states at the surface of the Mn_3_O_4_ cathode were explored by XPS and the total spectrum of XPS is shown in Figure S6 (Supporting Information), which includes Mn *2p*, Mn *3s*, O *1s*, Zn *2p*, S *1s*, and C *1s* patterns. The XPS patterns of Mn *3s*, Mn *2p*, and O *1s* in different states are displayed in Figure 2e–g. The spacing between the two Mn *3s* peaks of the pristine Mn_3_O_4_ electrode is 5.52 eV, which corresponds to an average valence state of 2.71, and is well‐matched with Mn_3_O_4_ (Figure 2e). The corresponding Mn *2p* orbital confirms that the Mn is predominantly trivalent state, as well as some divalent state and a small amount of tetravalent state (Figure 2f). When initial discharged the Mn_3_O_4_ electrode, the Mn *3s* peaks display a bandgap of 6.13 eV, which corresponds to an average valence of 2.02. The Mn *2p* orbital shows it to be predominantly divalent state, containing traces of trivalent and tetravalent states. It suggests that Zn^2+^ and H^+^ are inserted into the Mn_3_O_4_ cathode and lower their valence. When first charged with the Mn_3_O_4_ electrodes, the bandgap of Mn *3s* decreases to 5.17 eV and corresponds to an average valence of 3.11, indicating that the Zn^2+^ and H^+^ are extracted from the electrode and partly converted to MnO_2_. More tetravalent Mn also occurs in the Mn *2p* orbitals with a corresponding decrease in trivalence and divalence. This hybrid structure of Mn_3_O_4_/MnO_2_ formed in the in situ state has a better self‐adaptive state and is expected to show excellent structural stability and reversibility for ion storage. When switching the electrode to a discharged state, its average valence is ∼2.29 (bandgap of 5.89 eV), indicating that Zn^2+^ and H^+^ are insertion into the MnO_2_ and Mn_3_O_4_ accompanied by a decrease in average valence of Mn. The distribution of different valence states in Mn *2p* also fits well with its average valence state. Figure 2g confirms that the core‐level spectra of O *1s* correspond to the typical components of the Mn‐O‐Mn (529.9 eV) bond for MnO_2_. Mn‐OH (531.3 eV) and H‐O‐H (532.0 eV) peaks were also detected, which are ascribed to MnOOH and residual water in the materials, respectively. As shown in Figures S7 and S8 (Supporting Information), Zn *2p* and S *2p* further confirm the distribution of elements during the reaction. The pristine electrode exhibits a Zn and S absentee status. When initials discharge the battery, Zn *2p* and S *2p* peaks appear, which indicates the formation of Zn_0.5_MnO_2_ and Zn_4_(OH)_6_(SO_4_)·5H_2_O (ZHS). At the first charge state, the peaks of Zn *2p* and S *2p* become weaker, indicating that Zn^2+^ is detached from Mn_3_O_4_ and dissolution of ZHS occurs. The enhancement of the two peaks upon second discharge indicates the occurrence of ionic insertion and the formation of ZHS.

Scanning electron microscopy (SEM) and its corresponding energy dispersive spectrometer (EDS) mapping reveal the microstructure and elements distribution of the electrode at 1^st^ charge and discharge states. In the charge state, the electrode displays a uniform and dense morphology. The elements of the electrodes are C, Mn, and O, where C comes from the substrate and Mn and O from the active materials of MnO_2_ and Mn_3_O_4_ (Figure S9, Supporting Information). When switching the cathode to 1^st^ discharge state, the electrode displays a regular morphology with homogeneous ZHS (Figure S10, Supporting Information). The elements ratios of Zn, S, and O are increased, which can be ascribed to that the formation of Zn^2+^/H^+^ insertion into the cathode as well as the ZHS formation in the battery discharge.

**Figure 3:**
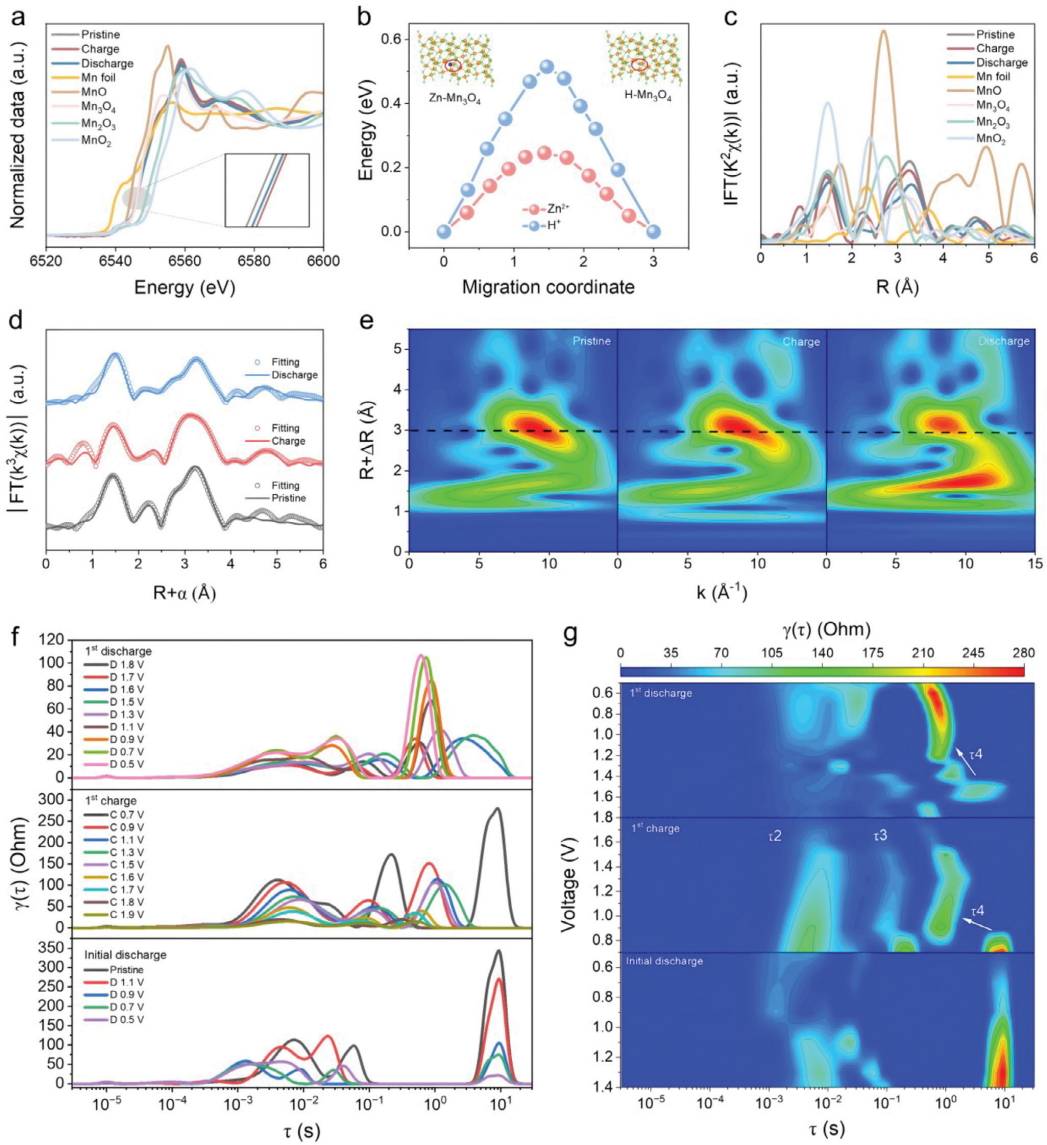
a) Normalized Mn K‐edge XANES spectra for pristine, charge, and discharge of Mn_3_O_4_, and standard samples. b) Migration energy barriers for Zn^2+^ and H^+^ in the (211) crystal plane of Mn_3_O_4_. c) FT‐EXAFS spectra for pristine, charge, and discharge of Mn_3_O_4_, and standard samples. d) EXAFS spectra and fitting curves for pristine, charge, and discharge of Mn_3_O_4_. e) Wavelet transform patterns for pristine, charge, and discharge of Mn_3_O_4_. f) DRT plot during charge–discharge process of Zn‐Mn_3_O_4_ coin cell. g) The contour plots of corresponding DRT at charge–discharge processes.

Apart from the above surface studies, the evolution of atomic structure and valence states in the bulk of Mn_3_O_4_ cathode were investigated by synchrotron X‐ray absorption spectroscopy. The X‐ray absorption near‐edge structure (XANES), Fourier transform‐extended X‐ray absorption fine structure (FT‐EXAFS) spectra, and Wavelet transforms were collected at the K‐edge of pristine, charged, and discharged Mn_3_O_4_ cathodes.^[^ ^42^, ^43^, ^44^, ^45^ ^]^ The XANES results show that the valence states of the electrodes are in the range of 2 to 3, with the order of being charged > discharged > pristine (**Figure** **3**a). Apparently, the valences of Mn with the discrepancy between XANES results and the XPS analysis, which may be ascribed to the different detection depths of these two different tests. The XPS test involves only depths from the surface down to a few nanometers, whereas XANES measures the entire electrode material. Specifically, the mass loading of Mn_3_O_4_ was ∼2 and 10 mg cm^−2^ for XPS and XANES test samples. The phase conversion of Mn_3_O_4_ to MnO_2_ and subsequent Zn^2+^/H^+^ co‐insertion/extraction chemistry mainly occurs in the electrode surface. Therefore, under high mass loading, a certain amount of Mn_3_O_4_ remains inside the electrode. This results in a relatively large amount of Mn_3_O_4_ species remaining in the bulk phase, reducing the average Mn valence state (Mn^2+^/Mn^3+^) measured by XANES. In contrast, XPS focuses on the surface‐active layer and preferentially reflects information on the high‐valence Mn species (Mn^2+^/Mn^3+^/Mn^4+^) involved in the electrochemical reaction. To elucidate this mechanism, we focused on the (211) crystal surface of Mn_3_O_4_, which corresponds to the most intense peak in the XRD pattern, and calculated the ion mobility energy barrier on this surface. The migration paths of Zn^2+^ and H^+^ on the C surface are shown in Figure S11 (Supporting Information). These results indicate that the surface diffusion energies (Figure 3b) of Zn^2+^ and H^+^ are significantly lower than their bulk diffusion energies (Figure 1c) within Mn_3_O_4_. It suggests that the energy storage of ions and the phase conversion are more likely to occur on the surface of the cathode. As a result, the highest valence state of the charged electrode is indisputable. The higher valence of the discharged electrode than the pristine electrode can be attributed to the formation of trivalent Zn_0.5_MnO_2_ and MnOOH on the electrode surface is more than Zn^2+^/H^+^ insertion into the Mn_3_O_4_.

The FT‐EXAFS spectrum of the pristine, charged, and discharged Mn_3_O_4_ electrodes exhibit the peak at 1.46–151 and 2.16–2.32 Å in R space, which corresponds to Mn─O and Mn─Mn coordination shells (Figure 3c).^[^ ^42^ ^]^ As illustrated in Figure 3d, the FT‐EXAFS spectrum is well‐fitted in R space using backscattering paths for Mn─O and Mn─Mn coordination in the electrodes. This is due to the reorganization of the Mn_3_O_4_ electrode during charging and discharging, resulting in changes in its bond length and coordination environment. Combined with the wavelet transform results as shown in Figure 3e and Figure S12 (Supporting Information), the charge state exhibits a slight shift toward lower R space than the pristine electrode. It can be ascribed to the change of coordination environment of Mn leads to a decrease in the Mn─O bond length and an increase in the electrostatic attraction to oxygen, increasing the valence of Mn.^[^ ^44^ ^]^ In the discharge state, the shift of R space is toward the higher direction, which is due to the Zn^2+^/H^+^ co‐insertion into the electrode, induces a large change in the coordination environment of Mn and some degree of lattice distortion, whereby the Mn─O bond is grown.

To further confirm the multistage chemistries in Zn‐Mn_3_O_4_ batteries, operando electrochemical impedance spectroscopy (EIS) measurements with distribution of relaxation times (DRT) analysis were systematically implemented. As shown in Figure S13a (Supporting Information), the charge transfer resistance (Rct) during the initial discharging exhibits the first increase (1.1 V) and then decrease (0.5–0.7 V) trend. This behavior arises from the activation of the initial phase of Zn^2+^/H^+^ insertion into Mn_3_O_4_ and the enhancement of the kinetics after activation. During the 1^st^ charging process, the Rct values gradually decreases with increasing charge voltage (Figure S13b, Supporting Information). This phenomenon stems from the fact that the practically structural transition of Mn_3_O_4_ to MnO_2_ optimizes the ion transport paths and provides additional active sites, and the kinetics of the Zn^2+^/H^+^ co‐insertion and subsequent Mn^2+^ deposition reactions are progressively enhanced. During the 1^st^ discharging process, localized accumulation of Zn^2+^/H^+^ extraction from the cathode induces pronounced concentration polarization, elevating ionic migration resistance and consequently Rct (Figure S13c, Supporting Information).^[^ ^46^ ^]^ DRT analysis of the pristine Zn‐Mn_3_O_4_ system revealed four characteristic peaks (τ1‐τ4) within the relaxation time range from 10^−6^ to 10^1^ s, corresponding to four distinct electrode processes (Figure S14, Supporting Information).^[^ ^47^ ^]^ Specifically, τ1 represents ohmic impedance, τ2 reflects Rct at the Zn anode redox interface, τ3 corresponds to the Rct in Mn_3_O_4_ cathode chemistry, while τ4 associates with ions’ diffusion impedance.^[^ ^48^, ^49^ ^]^ As shown in Figure 3f,g, dynamic analysis of peak evolution during charge/discharge cycles reveals the electrodes’ chemistries. During the initial discharging, Zn^2+^/H^+^ diffusion and Zn anode redox dominate the electrochemical process, while the near absence of τ3 is ascribed to the low capacity of the initial discharge.^[^ ^50^ ^]^ Upon the battery charging process, the stronger charge transfer resistance contribution at τ3 is due to the restricted Zn^2+^/H^+^ extraction kinetics. As the charging voltage increases, Mn_3_O_4_ occurs a practically phase transformation to MnO_2_ and Mn^2+^ deposition chemistry. Compared to the Zn^2+^/H^+^ extraction chemistry, the peaks of τ3 moves to a lower relaxation time, while the oxidation of Mn is more kinetically supported, resulting in a lower impedance contribution at τ3. When switching to discharging, the MnO_2_ dissolution (1.5–1.9 V) initially occurs, and the kinetics are stronger with weaker impedance contributions at τ3 and τ4. In the low voltage phase (0.5–1.4 V), the peak position is enhanced and shifted toward low relaxation times, which is consistent with the Zn^2+^/H^+^ insertion reaction (Figure S15, Supporting Information).

### 2.3 Electrochemical Performance of the Zn‐Mn_3_O_4_ Batteries

The optimization of the synthesis conditions of the Mn_3_O_4_ cathode was performed according to the electrochemical performance. We synthesized Mn_3_O_4_ by a hydrothermal method, with a strong emphasis on controlling hydrothermal time and temperature. First, the hydrothermal time is fixed at 12 h and the temperature range is from 110 °C to 180 °C. Figure S16 (Supporting Information) shows the long‐term cycling performance and charge/discharge curves of the constructed Zn‐Mn_3_O_4_ batteries at a current density of 0.5 A g^−1^. The Zn‐Mn_3_O_4_ battery maintains a specific capacity of 125 mAh g^−1^ after stable 700 cycles with Mn_3_O_4_ at a synthesis temperature of 120 °C. When the Mn_3_O_4_ hydrothermal reaction at 110 °C, 150 °C, and 180 °C, the Zn‐Mn_3_O_4_ batteries exhibit a lower initial specific capacity, and fast decay to lower than 20 mAh g^−1^ (Figure S16a, Supporting Information). Figure S16b–d (Supporting Information) displays the charge/discharge curves of the Zn‐Mn_3_O_4_ battery at different cycles. In the 1^st^ cycle, the battery shows a specific capacity of 70 mAh g^−1^ with Mn_3_O_4_ at a synthesis temperature of 110 °C, 86 mAh g^−1^ at a synthesis temperature of 120 °C, and lower than 20 mAh g^−1^ at a synthesis temperature of 150 °C and 180 °C. When switching to the 300^th^ and 700^th^ cycles, the battery with Mn_3_O_4_ cathode synthesis temperature at 120 °C always maintains the higher specific capacity over 120 mAh g^−1^, while lower 30 mAh g^−1^ at other temperatures. Figure S17a (Supporting Information) demonstrates the X‐ray diffraction (XRD) patterns of Mn_3_O_4_ obtained at different temperatures including 110 °C, 120 °C, and 150 °C. All three samples exhibit diffraction peaks corresponding to the standard card of Mn_3_O_4_ (JCPDS # 75–1560). The sharpness of the diffraction peaks increased significantly with increasing temperature, indicating an increase in crystallinity. The microscopic morphology shows the sample as nanowires at 110 °C (Figure S17b, Supporting Information), a mixture of nanowires and particles at 120 °C (Figure S17c, Supporting Information), and angular nanoparticles at 150 °C (Figure S17d, Supporting Information). It can be concluded that the Mn_3_O_4_ cathodes prepared at a hydrothermal temperature of 120 °C show a crystalline phase and microstructure suitable for ion storage with optimized electrochemical performance.

The hydrothermal reaction time also plays an important effect on the material formation and electrochemical properties. Thus, we fixed the optimized temperature of 120 °C and investigated the reaction times ranging from 2 to 24 h. Figure S18 (Supporting Information) exhibits the cycling performance and charge/discharge curves of the Zn‐Mn_3_O_4_ batteries with Mn_3_O_4_ cathodes constructed at different hydrothermal times. As illustrated in Figure S18a (Supporting Information), although the Mn_3_O_4_ cathode prepared by hydrothermal time for 2 h shows a high initial specific capacity (∼229 mAh g^−1^), its capacity completely decayed after 500 cycles. The battery with Mn_3_O_4_ under hydrothermal time of 12 h shows the best specific capacity play and stable cycling ability. The charge/discharge curves reveal the capacity exertion of the Zn‐Mn_3_O_4_ batteries under different cycles and further reveal that the optimal hydrothermal time is 12 h (Figure S18b–d, Supporting Information). The corresponding XRD patterns demonstrate poor crystallinity at a hydrothermal time of 2 h. While the diffraction peaks are sharper after hydrothermal times over 12 h, indicating that the crystals are better developed and increase with time (Figure S19a, Supporting Information). The SEM images exhibit that the Mn_3_O_4_ at 2 h is nanowire clusters states (Figure S19b, Supporting Information), while 12 h is a mixture of nanowires and particles (Figure S19c, Supporting Information), 16 h (Figure S19d, Supporting Information) and 24 h (Figure S19e, Supporting Information) are angular nanoparticles. Combined with the electrochemical performance, it can be concluded that Mn_3_O_4_ cathodes with a mixture of nanowires and particles as well as a well‐developed crystalline state are suitable for the storage of ions and exhibit excellent electrochemical performance.

**Figure 4:**
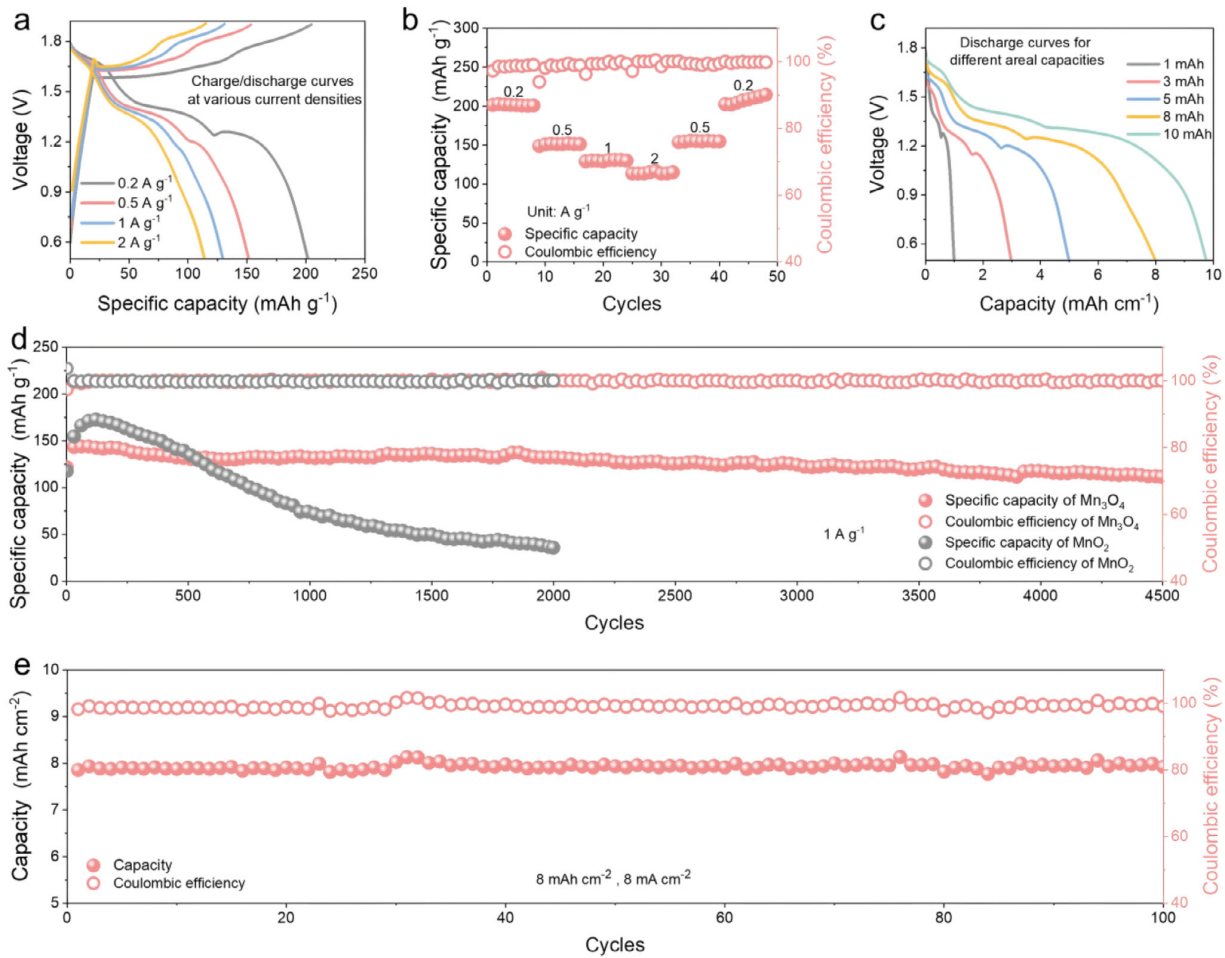
Electrochemical performance of the Zn‐Mn_3_O_4_ batteries. a) Charge/discharge curves at various current densities from 0.2 to 2 A g^−1^. b) Rate capacities at various current densities from 0.2 to 2 A g^−1^. c) Discharge curves at various areal capacities. d) Long‐term cycling performance of Zn‐Mn_3_O_4_ and Zn‐MnO_2_ batteries. e) Long‐term cycling performance of the Zn‐Mn_3_O_4_ battery at an areal capacity of 8 mAh cm^−2^.

The optimized hydrothermal condition for the Mn_3_O_4_ cathode is eventually held at 120 °C for 12 h. Subsequently, we systematically explore the electrochemical behaviors of the Mn_3_O_4_ under these optimized conditions. **Figure** **4**a shows the charge/discharge curves of the Zn‐Mn_3_O_4_ battery at different current densities. The battery exhibits three distinct discharge platforms, which correspond to the MnO_2_ dissolution and Zn^2+^/H^+^ ionic insertion chemistries. The battery displays a specific capacity of 202 mAh g^−1^ at 0.2 A g^−1^ and 114 mAh g^−1^ even at a high rate of 2 A g^−1^. Figure 4b exhibits the rate capacities of the Zn‐Mn_3_O_4_ battery. It always maintains a Coulombic efficiency (CE) near 100%, while keeping excellent specific capacity play at different current densities with good recovery performance. We evaluated the energy density of the Zn‐Mn_3_O_4_ battery, which is ∼226.9 Wh kg^−1^ based on the weight of the active material involved in the reaction (see experimental details). Excitingly, the Zn‐Mn_3_O_4_ battery achieves the largest areal capacity of 10 mAh cm^−2^ accompanied by a discharge voltage of ∼1.4 V (Figure 4c). The batteries at various areal capacities always maintain a higher CE of over 98%, indicating their great potential for development high energy density battery. Benefiting from the partial phase conversion of Mn_3_O_4_ cathode to MnO_2_ in the first charging. The formed hybrid Mn_3_O_4_/MnO_2_ processes the excellent structure stability and reversibility for Zn^2+^/H^+^ co‐insertion/extraction. In addition, the cathode provides an additional active area for Mn^2+^/MnO_2_ chemistry, expanding the capacity of the battery. As shown in Figure 4d, the Zn‐Mn_3_O_4_ battery exhibits a highly stable 4500 cycles at 1 A g^−1^, while maintaining a capacity retention of ∼78%. For comparison, the Zn‐MnO_2_ battery with commonly used detra‐MnO_2_ cathode shows a rapid capacity decay, falling below 100 mAh g^−1^ after 750 cycles and continuing to decay rapidly. To further investigate the reversibility of the battery at high areal capacities, we performed long‐term cycling measurements on Zn‐Mn_3_O_4_ batteries at different areal capacities. At an areal capacity of 1 mAh cm^−2^ and a current density of 2 mA cm^−2^, it displays a stable 1600 cycles (Figure S20, Supporting Information). While displays 550 cycles at 3 mAh cm^−2^ and over 300 cycles at 5 mAh cm^−2^ (Figures S21 and S22, Supporting Information). Excitingly, the Zn‐Mn_3_O_4_ battery still shows long‐term stability of 100 cycles at an areal capacity of 8 mAh cm^−2^ (Figure 4e) and shows over 40 cycles even at an areal capacity of 10 mAh cm^−2^ (Figure S23, Supporting Information). This implies that the Mn_3_O_4_/MnO_2_ hybrid cathode formed by the phase transition inherits the advantages of both materials, while plays excellent stability and reversibility even at high areal capacities.

The microscopic morphology of the Mn_3_O_4_ cathodes after cycling can provide further evidence of its structural stability. As shown in Figure S24 (Supporting Information), the SEM images of the pristine Mn_3_O_4_ electrode displays a uniform and dense surface morphology, indicating strong adhesion of the active material to the carbon fiber collector. Upon initial discharge, the cathode surface develops a dense lamellar structure, which remains consistent at the 1^st^, 5^th^, and 10^th^ discharge cycles (1^st^ D, 5^th^ D, and 10^th^ D). This structure is attributed to the formation of ZHS which occurs due to the insertion of H^+^ into MnO_2_ and Mn_3_O_4_. The subsequent increase in OH^‐^ concentration at the electrode interface promotes the formation of ZHS. At the corresponding charge states of 1^st^ C, 5^th^ C, and 10^th^ C, the Mn_3_O_4_ cathode displays a uniformly dense morphology, closely resembling the pristine electrode. This indicates that the Mn_3_O_4_ cathode remains structurally intact throughout cycling, demonstrating its excellent reversibility.

### 2.4 Scale‐Up Zn‐Mn_3_O_4_ Batteries for Practical Energy Storage Applications

**Figure 5:**
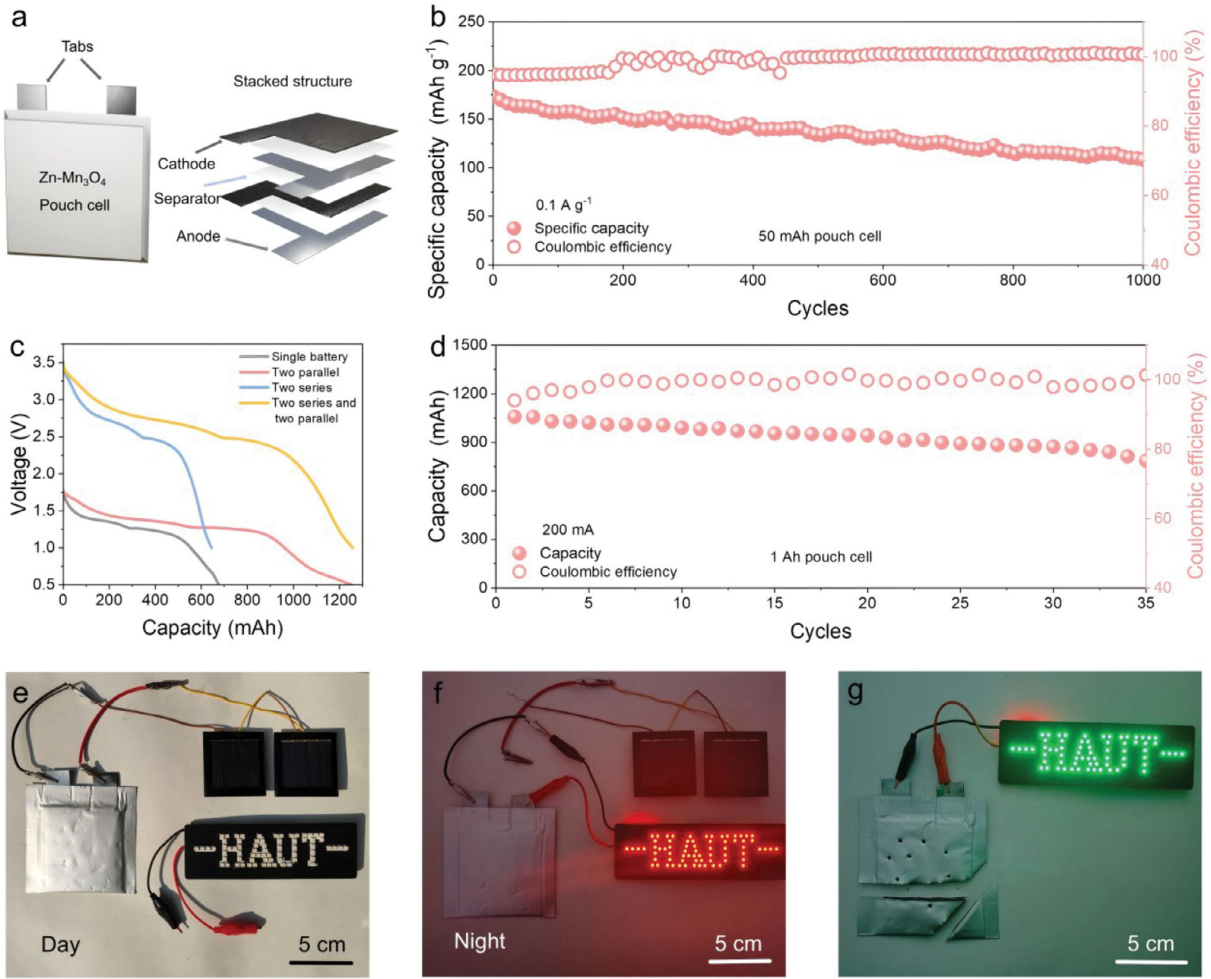
Electrochemical performance of the Zn‐Mn_3_O_4_ pouch cells. a) Schematic diagram of the Zn‐Mn_3_O_4_ pouch cell. b) Long‐term cycling performance of the Zn‐Mn_3_O_4_ pouch cell. c) Discharge curves of pouch cells with 650 mAh in different connections. d) Long‐term cycling performance of the Ah‐level pouch cell. e) Solar‐powered pouch cell energy storage system during the day. f) Solar‐powered pouch cell lighting up LEDs at night. g) Solar‐powered pouch cell lighting up LEDs after cutting and needling.

The excellent performance of Zn‐Mn_3_O_4_ batteries has prompted us to explore the possibility of large‐scale energy storage applications in their scaled‐up state. In this regard, we constructed an enlarged Zn‐Mn_3_O_4_ pouch cell with two pairs of electrodes used for stacking (**Figure** **5**a). Specifically, we assembled a 50 mAh pouch cell with dimensions of 4 × 4 cm^2^. The Zn‐Mn_3_O_4_ pouch cell exhibited an initial specific capacity of 173 mAh g^−1^ at a current density of 0.1 A g^−1^. After 1000 cycles, the specific capacity remained at 108 mAh g^−1^, corresponding to a capacity retention rate of 62.4% and an average Coulombic efficiency of 99.04% (Figure 5b). To expand the capacity, the size of the electrodes was further enlarged to ∼50 cm^2^, and the pouch cell capacity was increased to 200 mAh. The pouch cell demonstrated a stable cycling performance of more than 350 cycles (Figure S25, Supporting Information). Moreover, we further scaled up the capacity of a single pouch cell to 650 mAh and explored different configurations of series and parallel connections at a current density of 200 mA. As shown in Figure 5c, we performed series and parallel connections of the pouch cells and collected the charge/discharge curves. A battery module with two pouch cells connected in series exhibited a discharge voltage of ∼2.6 V and a discharge capacity of 650 mAh. In contrast, two pouch cells connected in parallel maintained a discharge voltage of 1.4 V and achieved a discharge capacity of 1250 mAh. Notably, the parallel‐connected 1 Ah pouch cell demonstrated a stable cycling performance of 35 stable cycles (Figure 5d). The excellent performance of the large‐capacity Zn‐Mn_3_O_4_ pouch cell provides us with the confidence to develop practical Zn batteries. We evaluated the practical energy density of the Zn‐Mn_3_O_4_ pouch cell, which is ∼54 Wh kg^−1^ based on the weight of the entire pouch cell (Figures S26 and S27, Supporting Information, see Experimental Details).

Furthermore, we conducted a preliminary exploration of the practical energy storage applications for Zn‐Mn_3_O_4_ pouch cells. As illustrated in Figure 5e, two 2 V photovoltaic panels were connected in parallel to charge a 50 mAh Zn‐Mn_3_O_4_ pouch cell for 2 h during the daytime. The solar‐powered battery module successfully powered a 1.5 V LED display at night (Figure 5f), demonstrating the stable charging capability of the Zn‐Mn_3_O_4_ battery using solar energy. This successful integration of Zn‐Mn_3_O_4_ pouch cells with photovoltaic panels highlights their great potential for future smart grid energy storage applications. In addition, we evaluated the safety performance of the Zn‐Mn_3_O_4_ pouch cell through pinning and shearing tests. The pouch cell demonstrates extremely high safety in the pinning and cutting tests, with no smoke or fire detected. In addition, the Zn‐Mn_3_O_4_ pouch cell after violent disintegration also outputs a stable voltage and lights up the LEDs for a long time(Figure 5g).

## 3 Conclusion

In this work, we revisit the energy storage mechanism of the Mn_3_O_4_ and demonstrate an energetic Zn‐Mn_3_O_4_ battery with ultrahigh areal capacity and long cycle lifespan. During the first charging process, the Mn_3_O_4_ cathode undergoes a practical phase conversion, forming a hybrid Mn_3_O_4_/MnO_2_ structure. This hybrid cathode not only retains the structural stability and high reversibility of Zn^2+^/H^+^ insertion/extraction but also introduces additional active sites for Mn^2+^/MnO_2_ reaction, greatly enhancing the areal capacity. As a result, the Zn‐Mn_3_O_4_ cion cell demonstrated a stable 4500 cycles, while inheriting its cycling stability at a high areal capacity of 8 mAh cm^−2^. Excitingly, the enlarged Zn‐Mn_3_O_4_ pouch cell displayed a stable 1000 cycles with 85% capacity retention. Further scaling up to a 1000 mAh pouch cell showed high efficiency and stable cycling with a real‐world energy density of ∼54 Wh kg^−1^. Moreover, the enlarged pouch cell successfully integrated with solar panel charging while exhibiting excellent safety. The design of a novel MNO cathode with in situ phase conversion provided a strategy for the development of energetic Zn‐MNO batteries for practical applications.

## 4 Experimental Section

### Materials

All chemical reagents and materials were used directly without treatment. Potassium permanganate (KMnO_4_, ≥99%), Ethanol absolute (CH_3_CH_2_OH, ≥99.5%), Zinc sulfate heptahydrate (ZnSO_4_·7H_2_O, ≥99%), Manganese sulfate monohydrate (MnSO_4_·H_2_O, ≥99%) N‐methyl‐2‐pyrrolidone (NMP) from Sinopharm Chemical Reagent Co., Ltd. Zn foil (thickness of 50 µm) was provided by Beijing Xinruichi Technology Co., Ltd. Whatman glass fiber (GF/B, thickness of 675 µm) and Carbon cloth (standard: 30 g m^−2^) were purchased from Alibaba Group. Polyvinylidene fluoride (PVDF) from Wah Hsin Plastic Co., Ltd. Ketjen black EC‐600JD from Zhengzhou Jinghong Battery Material Co., Ltd.

### Synthesis of Mn_3_O_4_ Cathode

The Mn_3_O_4_ cathode material was synthesized through a one‐step hydrothermal method. Specifically, 1 g of KMnO_4_ was dissolved in a mixture solution consisting of 30 mL of H_2_O and 50 mL of CH_3_CH_2_OH. Subsequently, the completely dissolved mixture was transferred to a 100 mL polytetrafluoroethylene‐lined autoclave. The hydrothermal temperatures were set at 110 °C, 120 °C, 150 °C, and 180 °C, while the reaction times were 2, 8, 12, 16, and 24 h. After the hydrothermal reaction was completed, the samples were centrifuged three times with deionized water, and then dried in an oven at 70 °C for 12 h. Finally, the samples were ground to obtain the Mn_3_O_4_ cathode material.

### Battery Fabrication

Mn_3_O_4_, Ketjen black, and PVDF were uniformly mixed and grind in a ratio of 7:2:1. Subsequently, adding appropriate NMP and grinding for 30 min to form a slurry. Thereafter, the slurry was evenly scraped onto carbon cloth and then dried at 80 °C for 12 h to obtain the Mn_3_O_4_ cathode electrodes. In preparation for the Zn‐Mn_3_O_4_ coin cell, the thickness of the scraped electrodes was 300 µm, and the mass loading of the active substance was ∼3.4 mg cm^−2^. Then the electrode was cropped with a diameter of 12 mm, along with a Zn foil with a diameter of 12 mm as the anode, and 675 µm glass fiber with a diameter of 16 mm as the separator. The electrolyte was a hybrid of 2 M ZnSO_4_ and 0.2 M MnSO_4_ with a dosage of 80 µL. High areal capacity Zn‐Mn_3_O_4_ batteries were assembled and tested using a homemade plexiglass devices with an effective electrode reaction area of 1 cm^2^ and 3 mL of electrolyte. For cathodes with capacity of 3 mAh and below, the slurry was coated onto both sides of the carbon cloth current collector using a doctor blade with a thickness of 1000 µm. The mass loading of the active material on the electrode sheet was ∼25 mg cm^−2^. Cathodes with capacities of 5, 8, and 10 mAh were fabricated by stacking multiple individual cathodes. Specifically, the 5 mAh cathode was comprised of two stacked pieces, and the 8 mAh cathode was comprised of three‐pieces, and the 10 mAh cathode was comprised of four‐pieces. Notably, the individual cathodes were stacked while the slurry was still wet to ensure tight adhesion after drying. This approach yielded mass loadings of ∼48, 70, and 96 mg cm^−2^ for the 5, 8 and 10 mAh cathodes, respectively.

In the Zn‐Mn_3_O_4_ pouch cell, the slurry was sandwiched with 4 cm × 4 cm (corresponding to 50 mAh pouch cell) and 7 cm × 7 cm (corresponding to 200 mAh pouch cell) carbon cloths, and the mass loading of the active substance was ∼20 mg cm^−2^. 7.5 cm × 7.5 cm glass fiber as the separator, and the electrolyte content was ∼30 mL. The 650 mAh pouch cell employs an electrode with a size of 7 cm × 7 cm. Stacking two such electrodes, each electrode has an active material loading of ∼36 mg cm^−2^.

### Materials Characterizations

Crystalline phase of the cathode was carried out through X‐ray diffraction (XRD, Smart Lab, Japan) with Cu‐Kα radiation. The surface composition and electronic states were probed by X‐ray photoelectron spectroscopy (XPS, Thermo ESCALAB 250Xi), using a monochromatic Al Ka source 1486.6 eV. Scanning electron microscopy (SEM, JEOL‐6700F) and its companion energy dispersive spectroscopy (EDS) were employed to observe the morphological structure and elemental distribution of the materials. X‐ray absorption spectroscopy data were collected at the BL14W1 station in Shanghai Synchrotron Radiation Facility (SSRF) to collect the electronic structure and the coordination environment around atoms of Mn_3_O_4_ cathodes.

### Electrochemical Measurements

Cyclic voltammetry (CV) and electrochemical impedance spectroscopy (EIS) were conducted through an electrochemical workstation (CHI608, Shanghai, China). The CV tests were conducted in a Zn‐Mn_3_O_4_ coin cell with a scan rate of 0.1–0.4 mV s^−1^ with a voltage range of 0.8–1.9 V versus Zn^2+^/Zn. The EIS was performed on Zn‐Mn_3_O_4_ coin cell under intermittent charge–discharge cycling. During the stepwise charge/discharge process, the EIS measurements were acquired across a frequency range of 0.1–100 kHz with an amplitude of 5 mV under open‐circuit potential conditions, when the voltage reached incremental steps of 0.1 V during either charging or discharging. To resolve the underlying electrochemical processes in the Zn‐Mn_3_O_4_ battery system, the distribution of relaxation times (DRT) was extracted from the EIS data.^[^ ^48^ ^]^ The DRT‐impedance, *Z_DRT_* (*f*), at a frequency *f*, is expressed as

(1) $$Z_{DRT}\left(f\right)=i2\pi fL_{0}+R_{\infty }+\int_{-\infty }^{+\infty }\frac{\gamma \left(\log\tau \right)}{1+i2\pi f\tau }d\log\tau$$

where *L_0_*, *R_∞_*, *τ*, and *γ(*log*τ)* denote the inductance, ohmic resistance, timescale, and DRT function, respectively. The total polarization resistance (*R_pol_*) was computed via:

(2) $$R_{pol}=\int_{-\infty }^{+\infty }\gamma \left(\log\tau \right)d\log\tau$$

The charge/discharge and cycling tests of the batteries were carried out using a battery test system (Neware, Shenzhen, China). The Zn‐Mn_3_O_4_ batteries were subjected to a constant current charge to a cutoff voltage of 1.9 V, followed by discharge at the same current rate down to 0.5 V. All electrochemical measurements were conducted at room temperature.

### Theoretical Calculations Details

The Vienna Ab‐initio Simulation Package (VASP) based on density functional theory (DFT) were employed to calculate the Mn_3_O_4_ as a cathode material. The calculations were based on the generalized gradient approximation (GGA) with the Perdew‐Burke‐Ernzerhof (PBE) exchange‐correlation functional. The projector augmented wave (PAW) method was utilized to describe the interaction between core and valence electrons, and a plane‐wave basis set with a cutoff energy of 400 eV was adopted. Geometry optimizations were performed using the Monkhorst‐Pack k‐points of 3 × 3 × 3. The convergence criteria were set to an electronic energy difference of 1 × 10^−4^ eV and a force convergence of −1 × 10^−4^ eV Å^−1^ to ensure the accuracy of the calculations. The calculations were performed to investigate the differential charge, migration barrier, adsorption energy, DOS, etc. The study of the migration energy barrier includes the bulk phase and surface of Mn_3_O_4_ cathodes.

### Calculation of the Energy Density

The size of the Mn_3_O_4_ cathode was 7 cm × 7 cm and the loading of active material was 2.22 g. The anode used a 7 cm × 7 cm Zn foil. Glass fibers of 0.5 mm thickness were used as separators and the electrolyte was 2 M ZnSO_4_ + 0.2 MnSO_4_. In the pouch cell, the weights of the cathode, anode, separator, electrolyte, and package were 3.16 g, 1.62 g, 0.29 g, 7.18 g, and 1.58 g, respectively (Figure S26, Supporting Information). The pouch cell displays a total discharge energy of ∼750 mWh (Figure S27, Supporting Information). Therefore, the calculated energy density was ∼54 Wh kg^−1^. Calculation of the energy density based on active materials. The Zn‐Mn_3_O_4_ battery exhibits a capacity of 0.68 mAh and a discharge energy of 1.11 mWh. The mass of the active material in the Mn_3_O_4_ cathode was 0.0034 g, and the mass of the Zn foil participating in the reaction at the anode was 0.000837 g. Consequently, the total mass of the active materials amounts to 0.004237 g. Based on the active materials, the energy density of the Zn‐Mn_3_O_4_ battery was calculated to be 226.9 Wh kg^−1^.

## Conflict of Interest

The authors declare no conflict of interest.

## Supporting information



Supporting Information

## Data Availability

The data that support the findings of this study are available from the corresponding author upon reasonable request.
