# Supplementary material for: From Charge Storage Rulebook Rewriting to Commercial Viability of Zinc‐Manganese Batteries
Source: Adv Sci (Weinh). 2025 Jul 2;12(37):e09520. doi: 10.1002/advs.202509520 (PMC12499474; doi:10.1002/advs.202509520)
Supplement: Supplementary file 1 — Supporting Information [file ADVS-12-e09520-s001.docx]

Supporting Information

**From Charge Storage Rulebook Rewriting to Commercial Viability of Zinc-Manganese Batteries**

Xinhua Zheng^[a]^, Bibo Han^[a]^, Shikai Liu^[a]*^, Shiya Huang^[a]^, Song Wu^[a]^, Mingyan Chuai^[b]*^, Faxing Wang^[c]*^, Yuping Wu^[c]*^

^[a]^ X. Zheng, B. Han, S. Liu, S. Huang, S. Wu
School of Materials Science and Engineering, Henan University of Technology
450001 Zhengzhou, Henan (China)
E-mail: shikai_liu@haut.edu.cn

^[b]^ M. Chuai
State Key Laboratory of Structural Chemistry, Fujian Institute of Research on the Structure of Matter, Chinese Academy of Sciences
350002, Fuzhou, Fujian (China)
E-mail: chuaimingyan@fjirsm.ac.cn

^[c]^ F. Wang, Y. Wu
Confucius Energy Storage Lab, School of Energy and Environment & Z Energy Storage Center, Southeast University
210096 Nanjing, Jiangsu (China)
E-mail: [faxing.wang@seu.edu.cn](mailto:faxing.wang@seu.edu.cn); wuyp@seu.edu.cn

Supporting information for this article is given via a link at the end of the document.


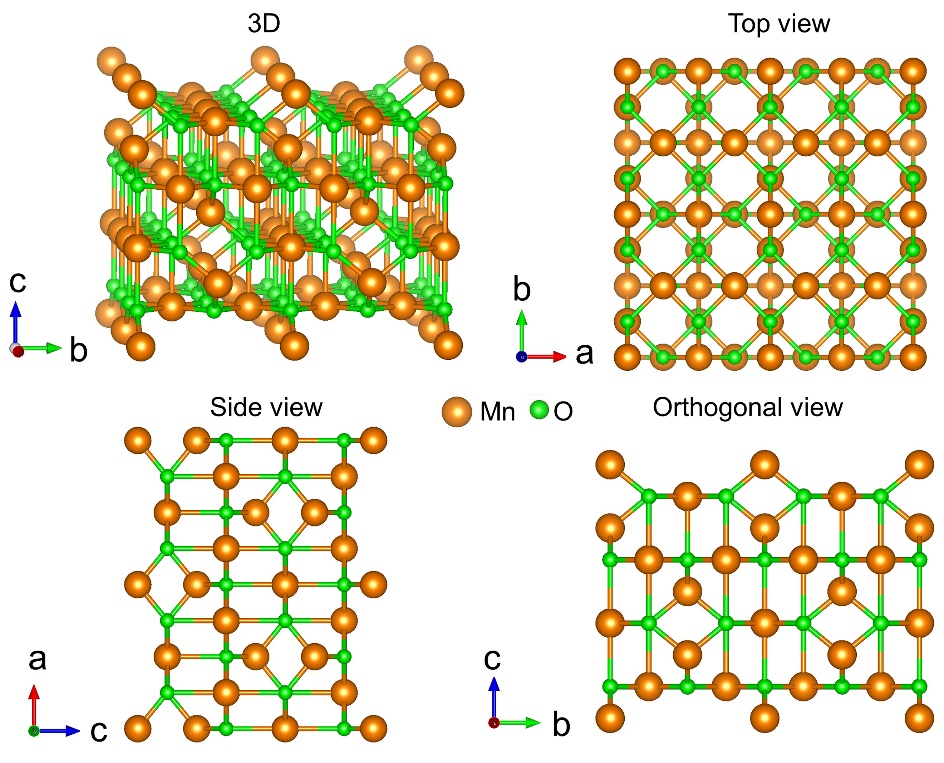


**Figure S1** The geometrical models of Mn_3_O_4_ at different views.


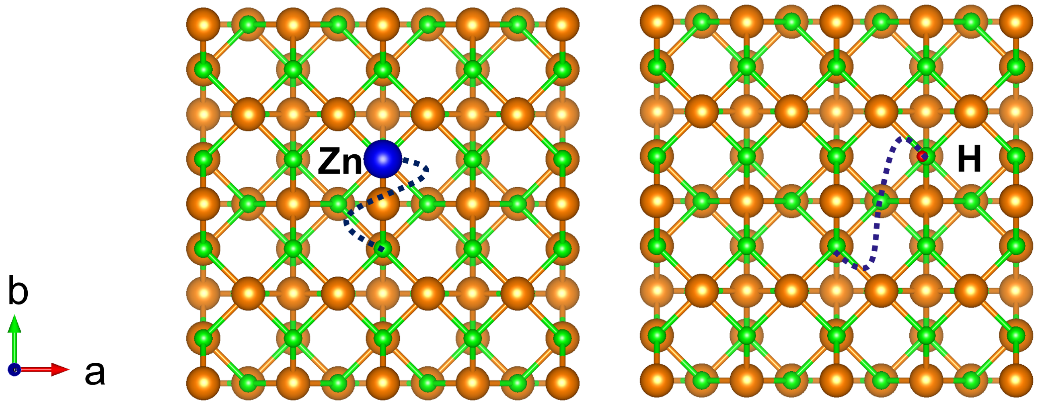


**Figure S2** The optimized Zn^2+^ and H^+^ diffusion pathway in Mn_3_O_4_.


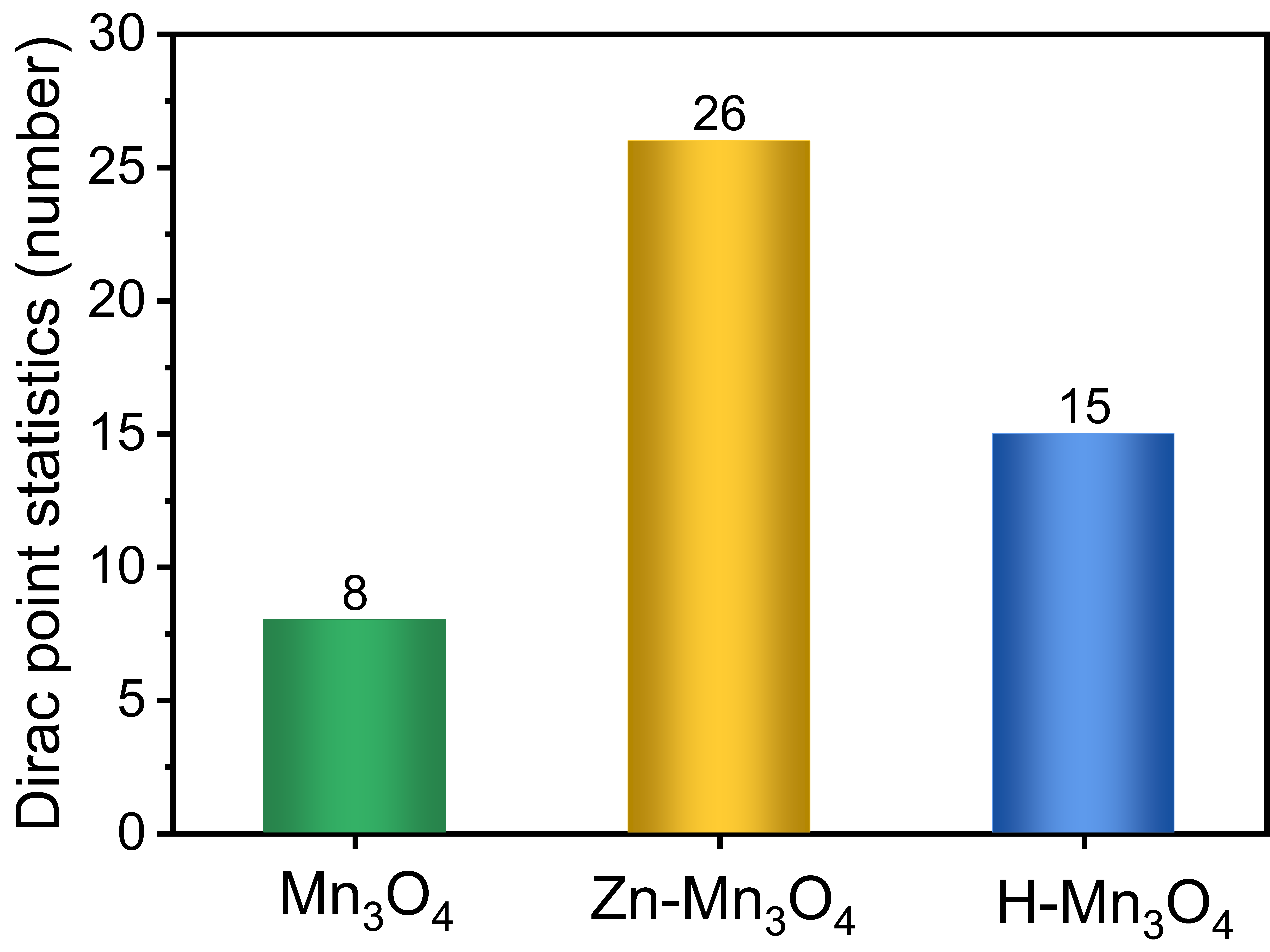


**Figure S3** Dirac point statistics for the Mn_3_O_4_, Zn-Mn_3_O_4_, and H-Mn_3_O_4_.


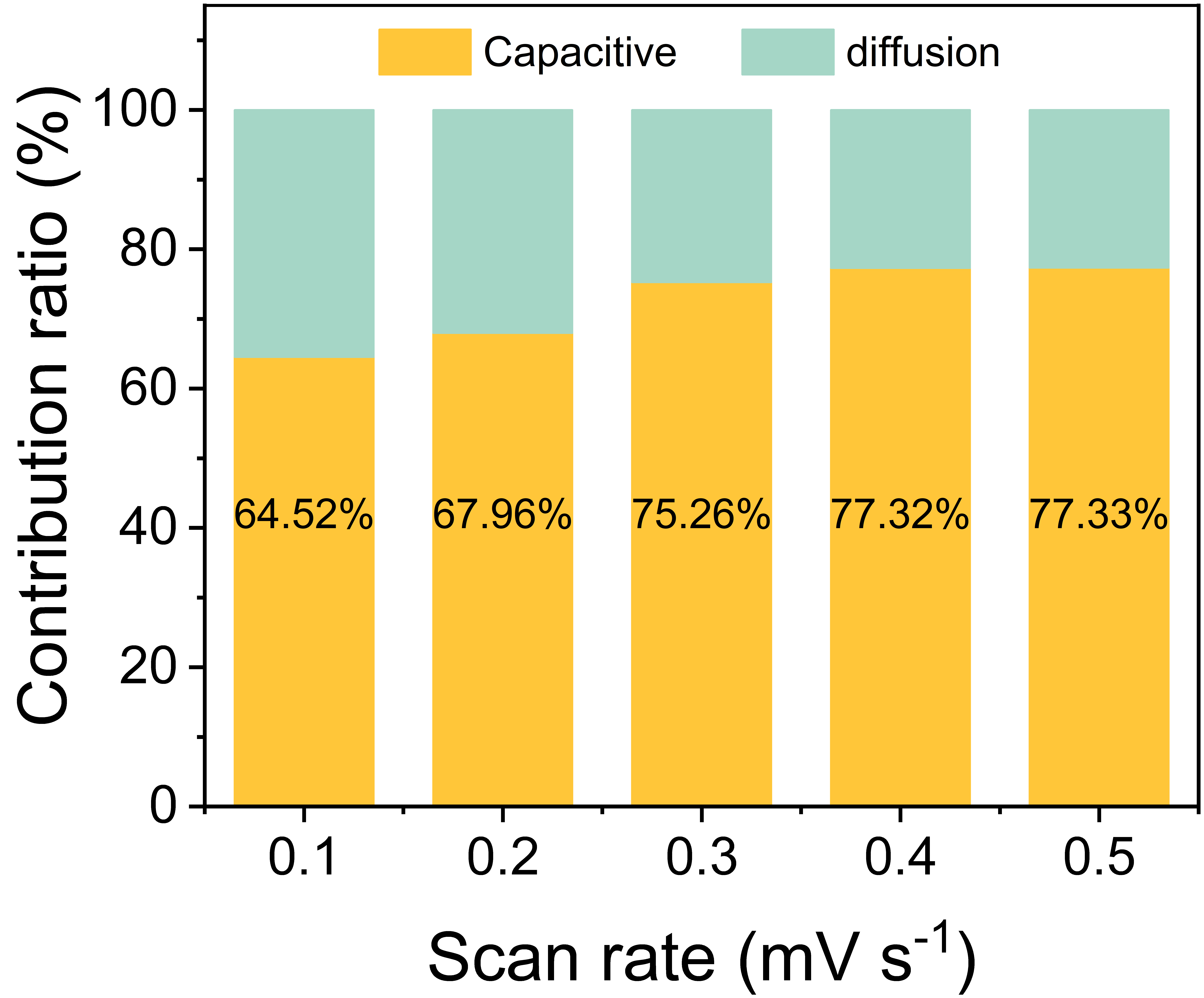


**Figure S4** Capacitive contribution to the total capacity.


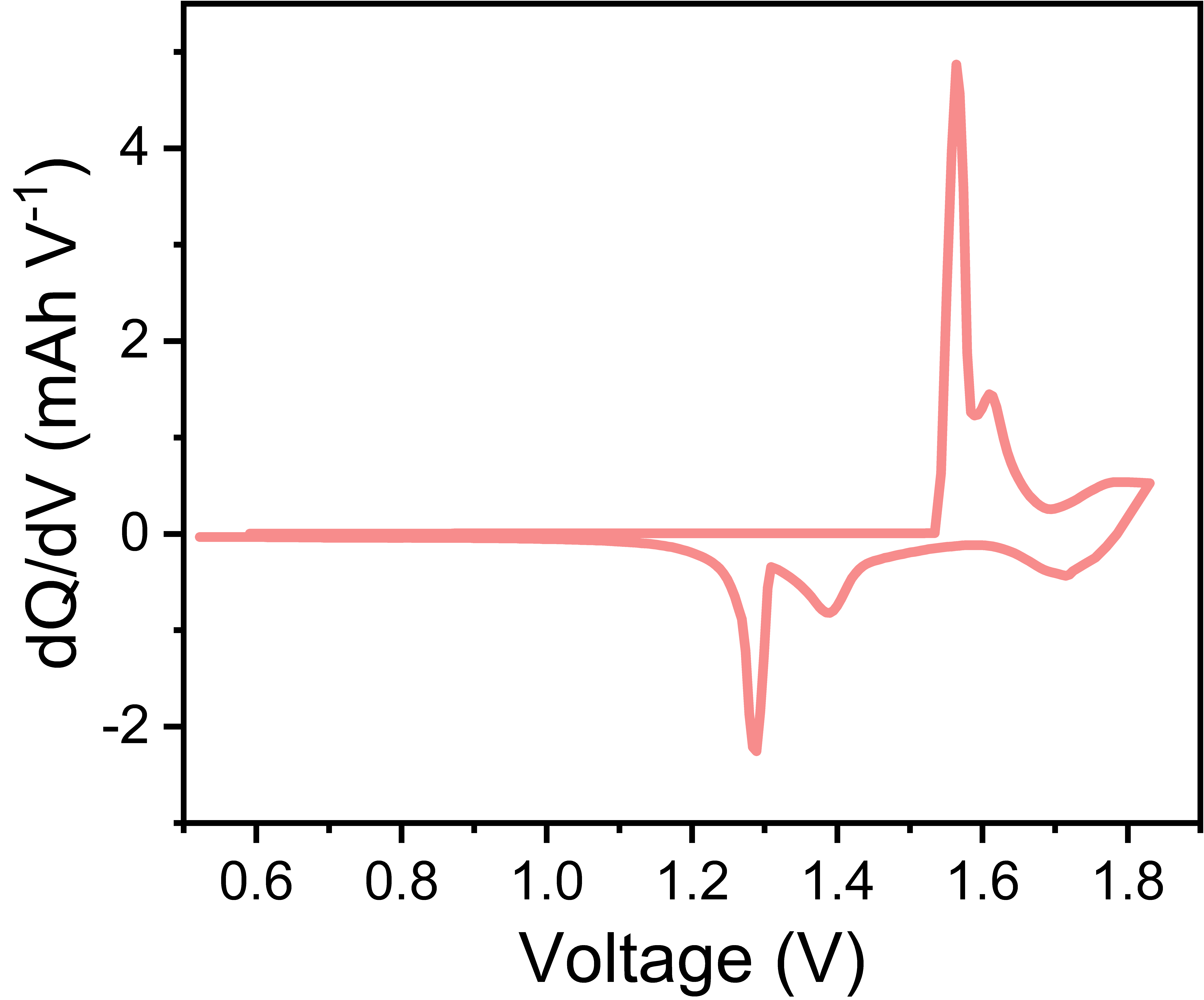


**Figure S5** The dQ/dV *vs.* voltage curve of Zn-Mn_3_O_4_ battery.


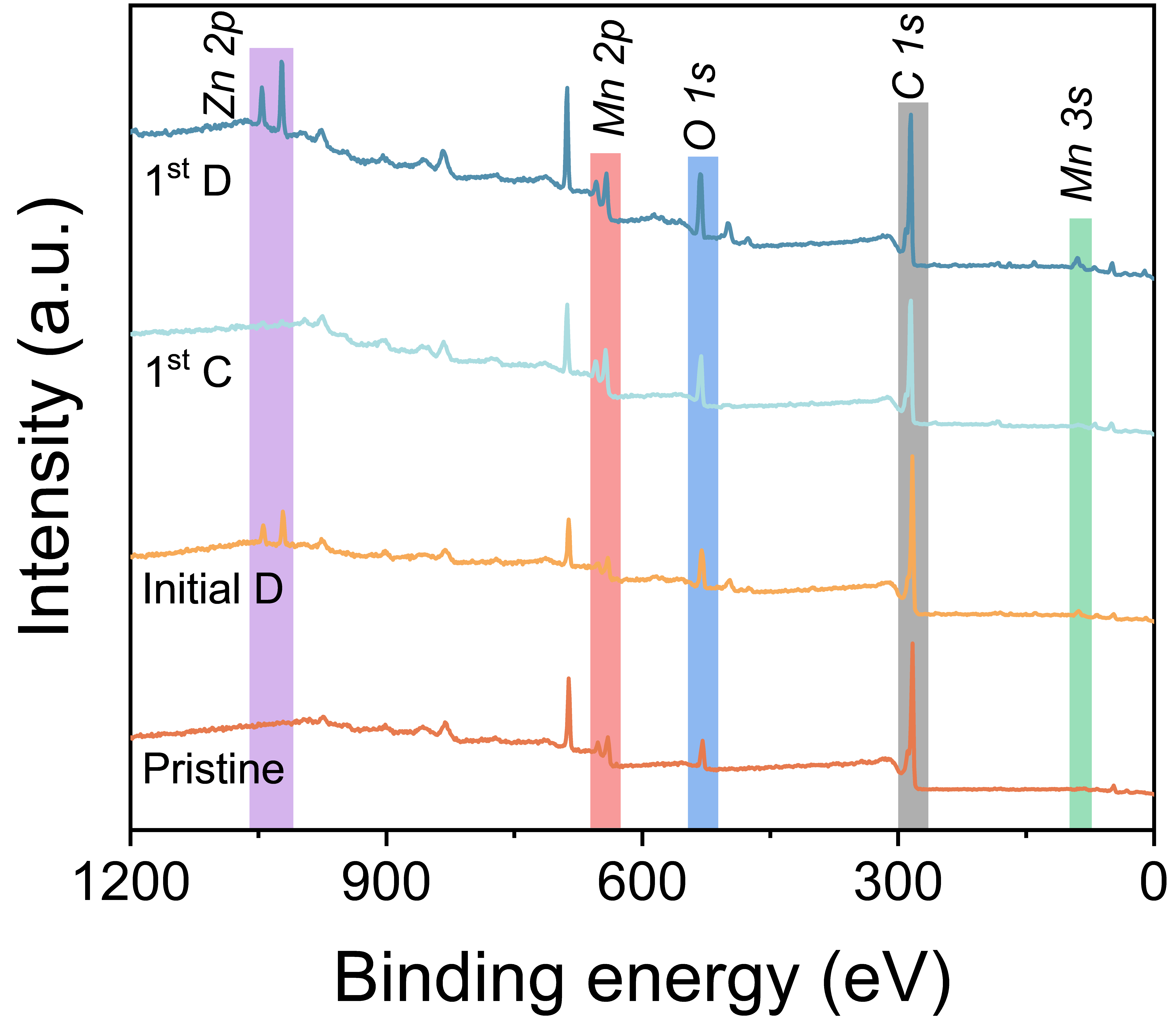


**Figure S6** XPS spectra of Mn_3_O_4_ at full spectrum, where “C” and “D” represent charge and discharge states, respectively.


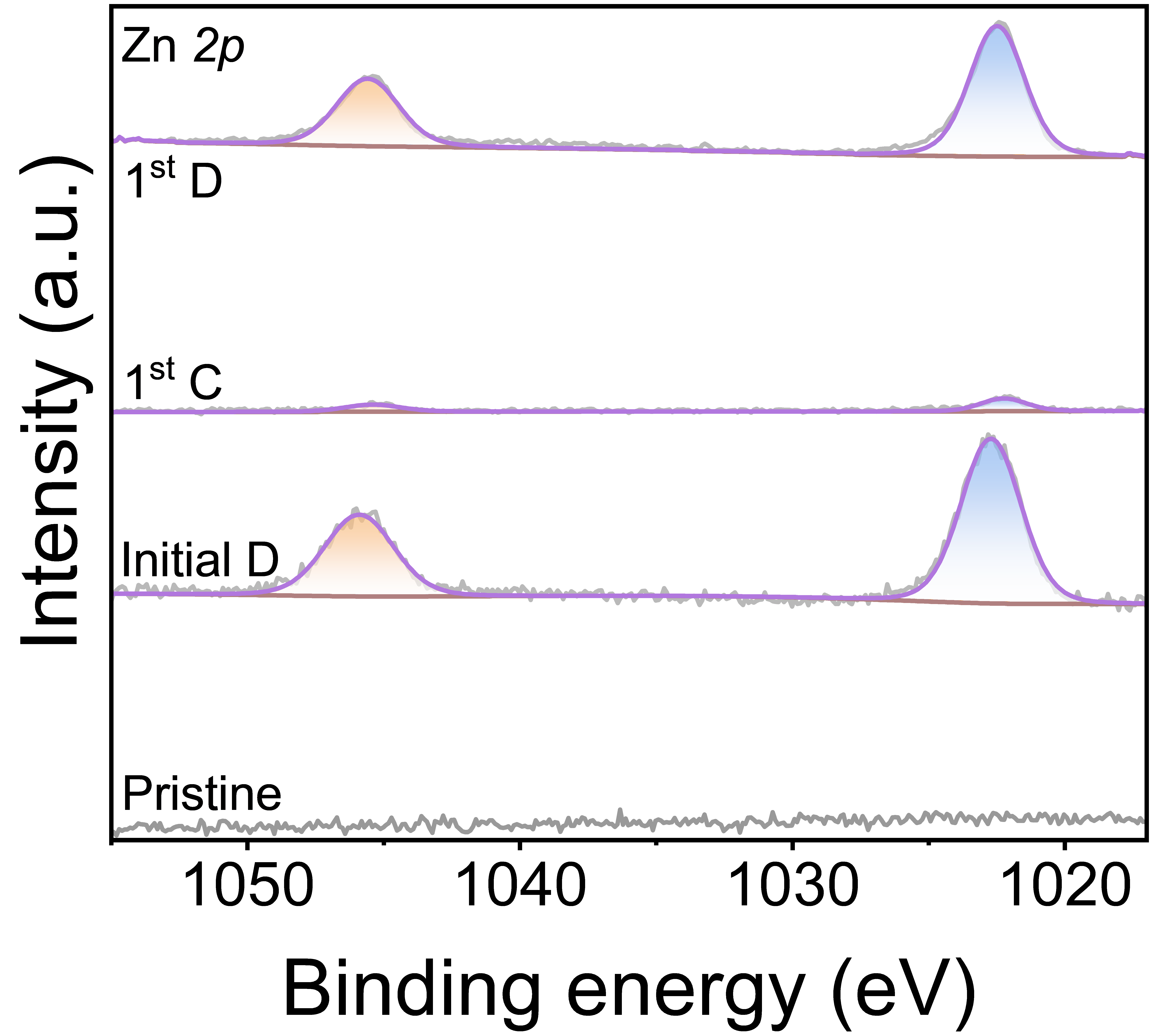


**Figure S7** XPS spectra of Mn_3_O_4_ at Zn *2p*, where “C” and “D” represent charge and discharge states, respectively.


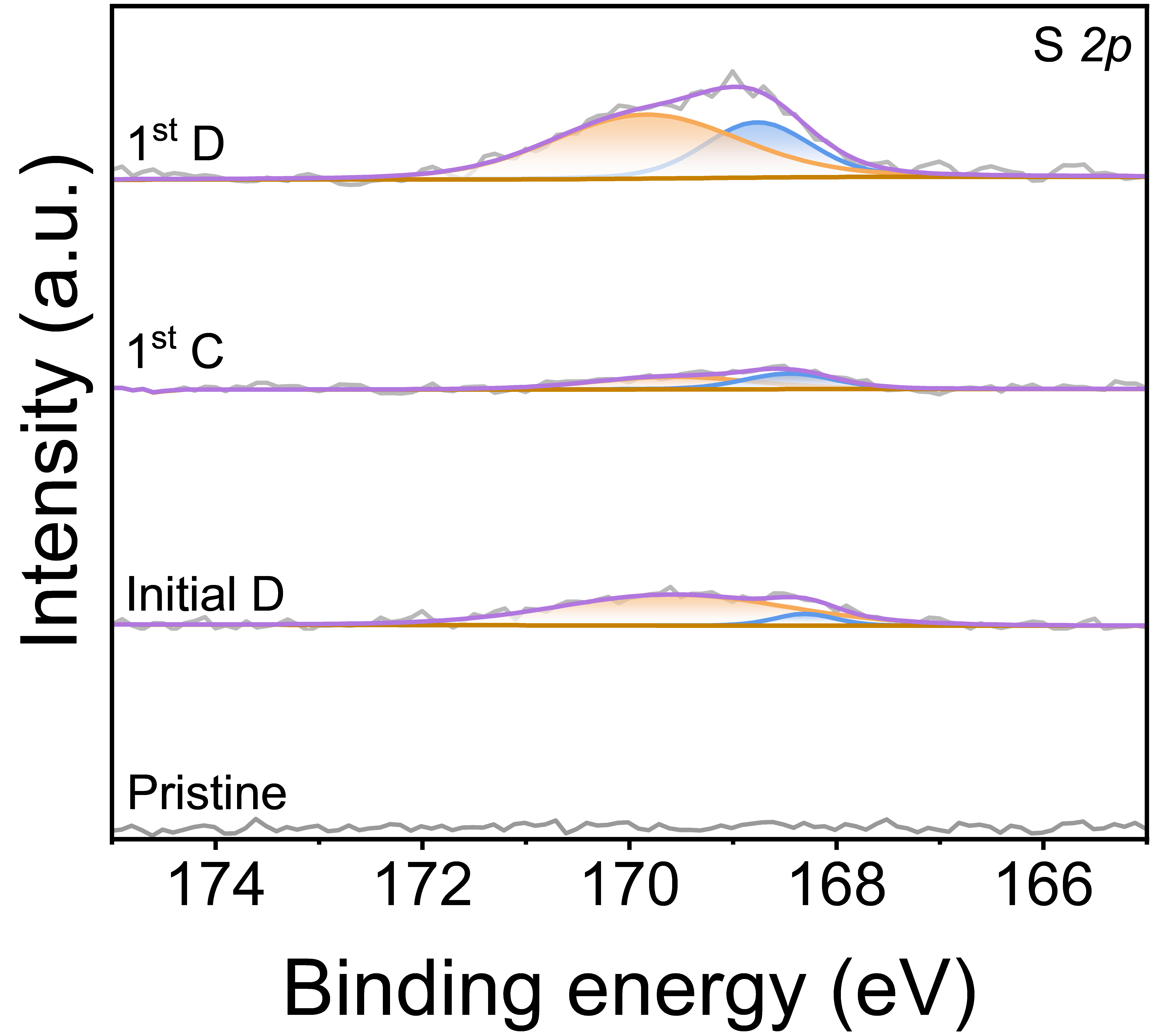


**Figure S8** XPS spectra of Mn_3_O_4_ at S *2p*, where “C” and “D” represent charge and discharge states, respectively.


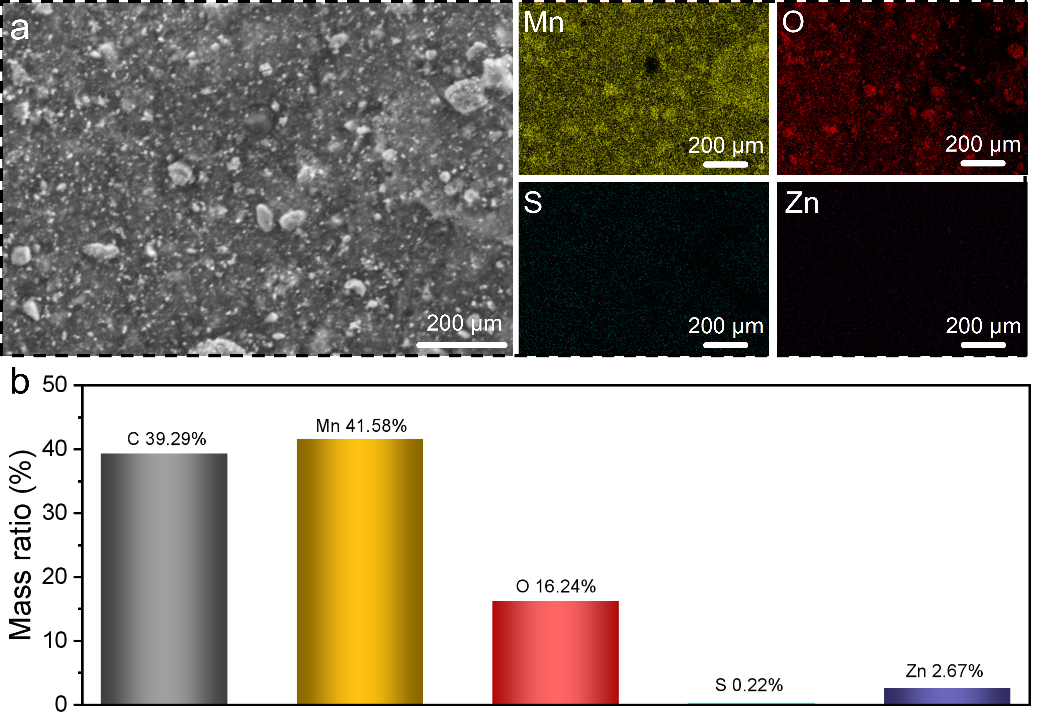


**Figure S9** Energy Dispersive Spectrometer mapping and mass ration distributions of the electrode at the 1^st^ charge states.


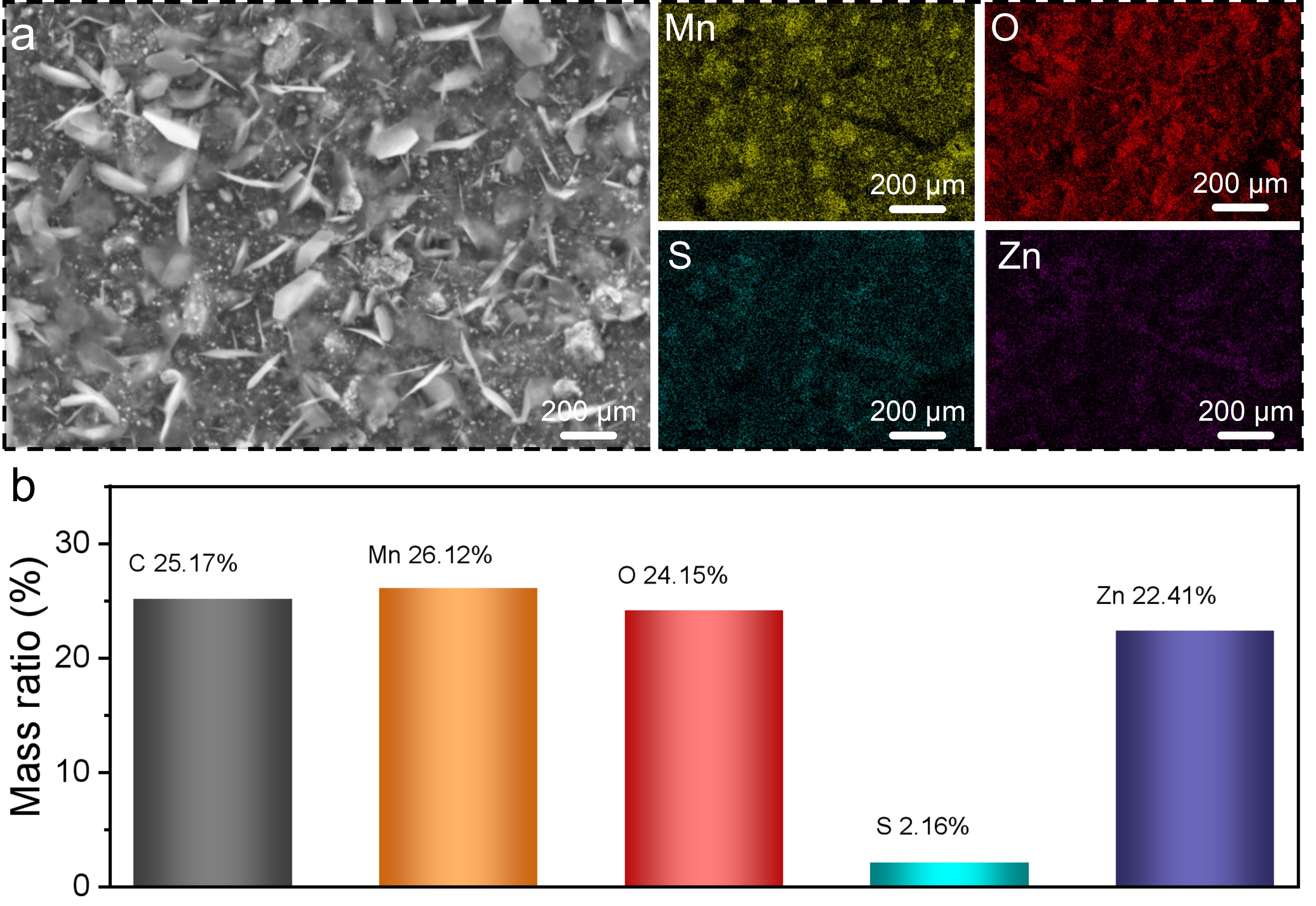


**Figure S10** Energy Dispersive Spectrometer mapping and mass ration distributions of the electrode at the 1^st^ discharge states.


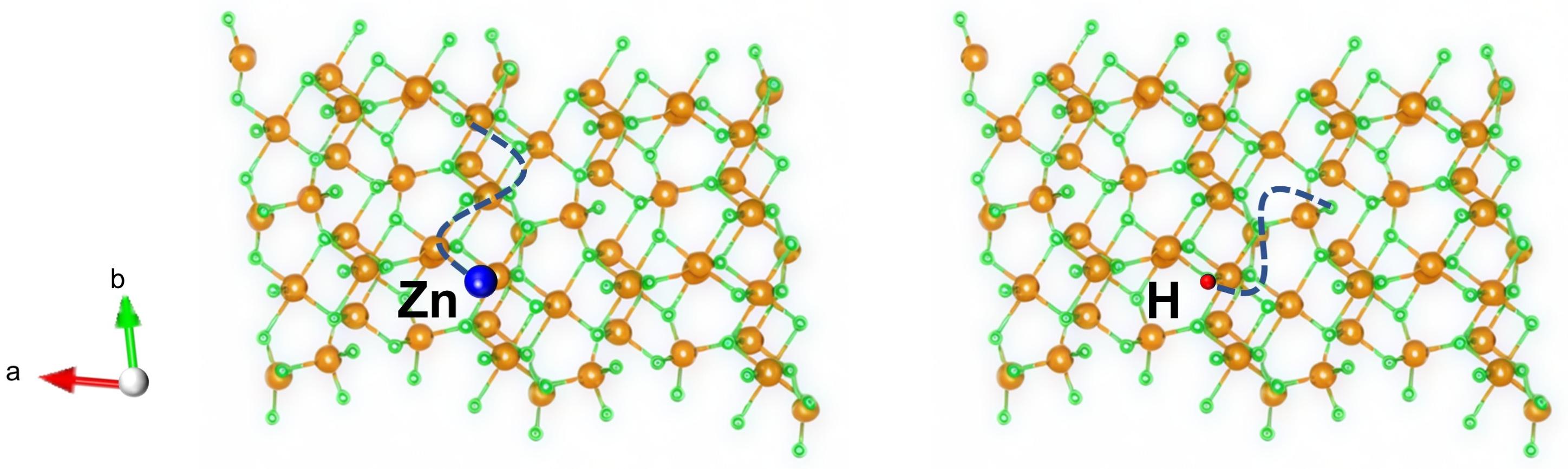


**Figure S11** Zn^2+^ and H^+^ diffusion pathway into the (211) surface of Mn_3_O_4_.


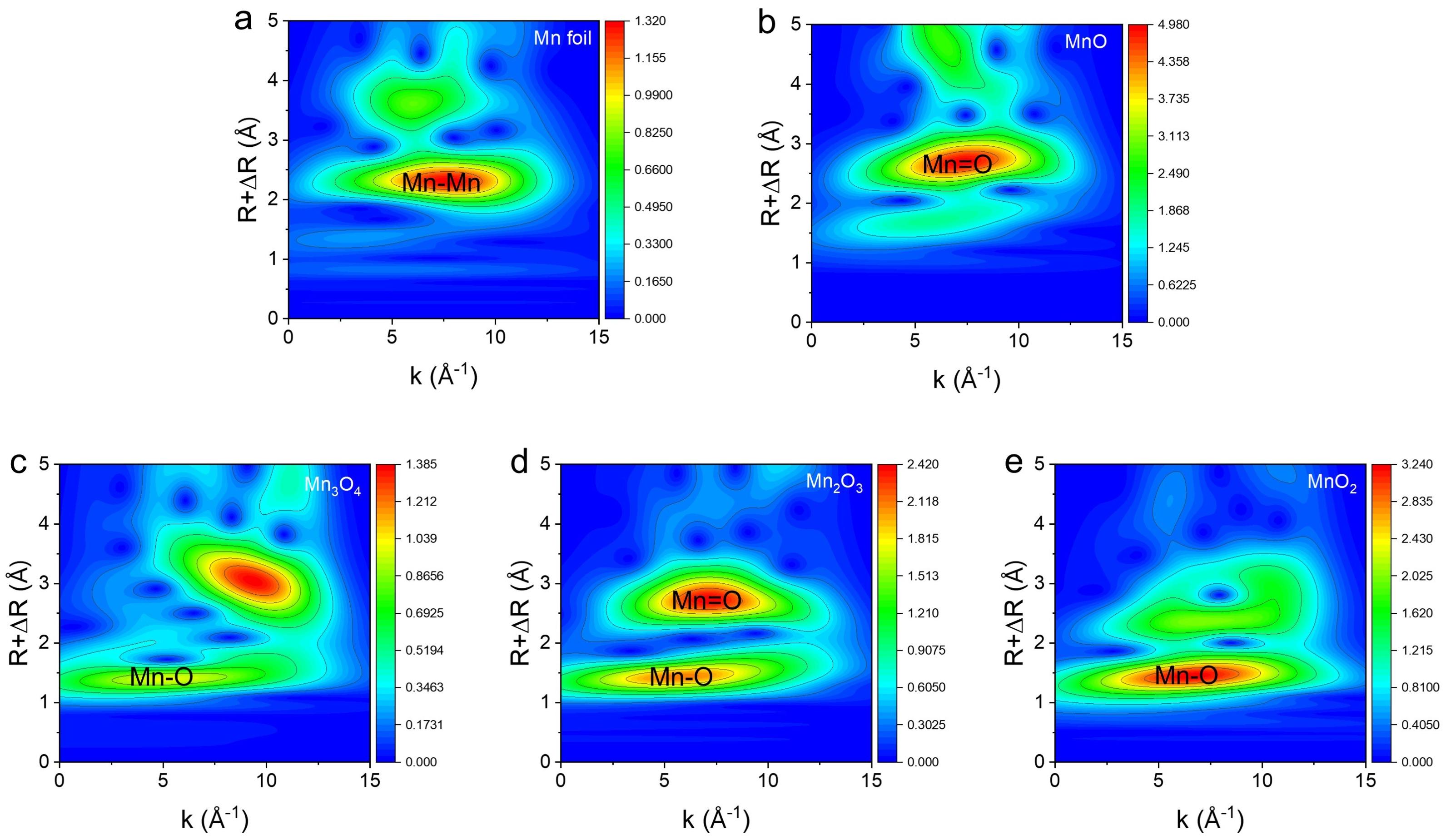


**Figure S12** Wavelet transform patterns of Mn foil, MnO, Mn_3_O_4_, Mn_2_O_3_ and MnO_2_.


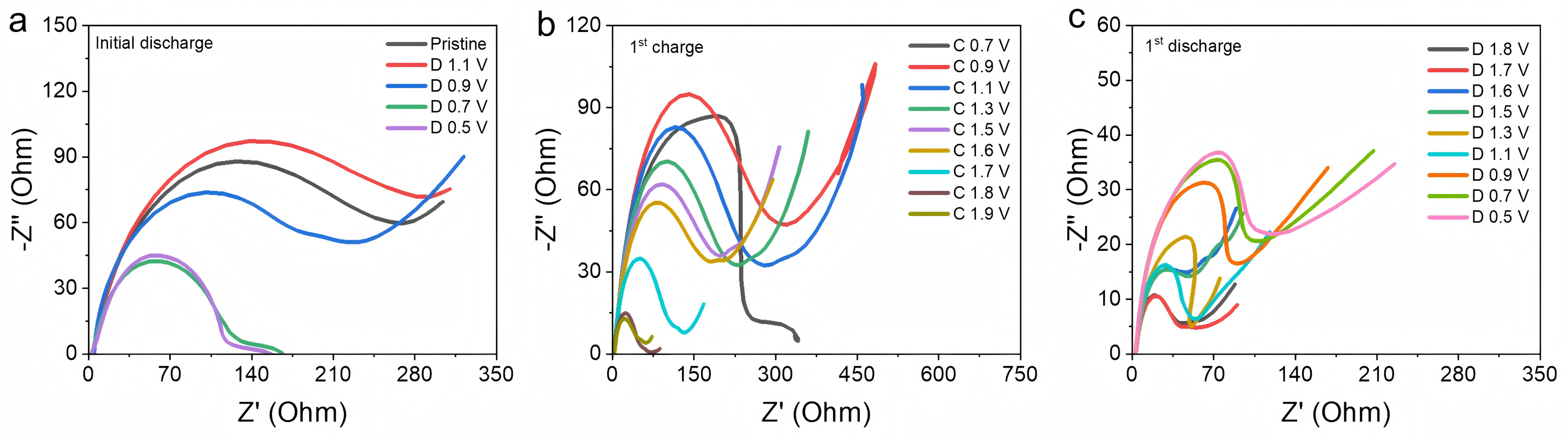


**Figure S13** The Nyquist plot of EIS during the charge-discharge process of Zn-Mn_3_O_4_ coin cell. (a) The first discharge stage. (b) The charging stage. (c) The second discharge stage.


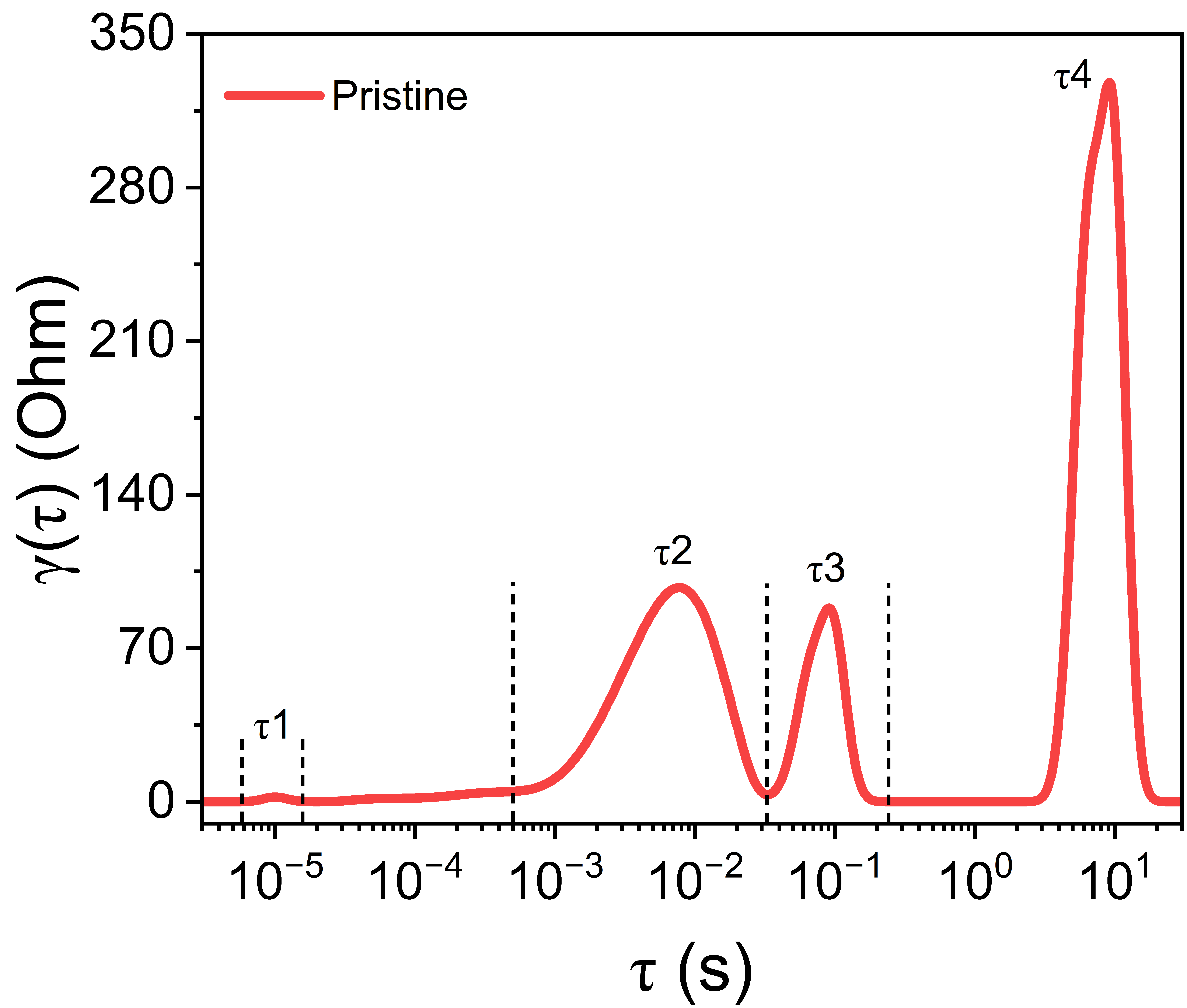


**Figure S14** DRT plot of the pristine state of the Zn-Mn_3_O_4_ coin cell.


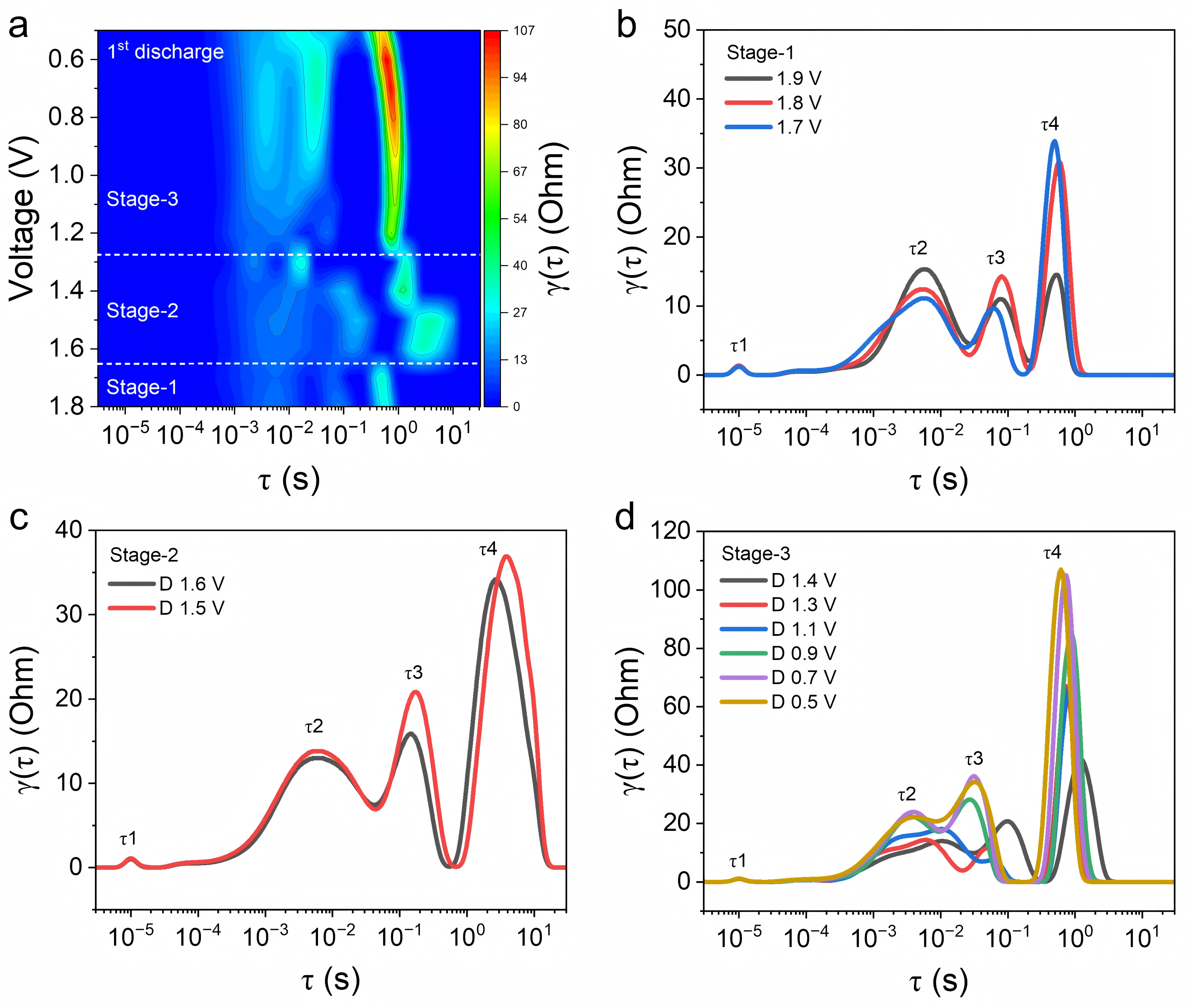


**Figure S15** (a) The contour plots of corresponding DRT at different stages during discharging. (b) DRT plots of stage-1 for discharge. (c) DRT plots of stage-2 for discharge. (d) DRT plots of stage-3 for discharge.


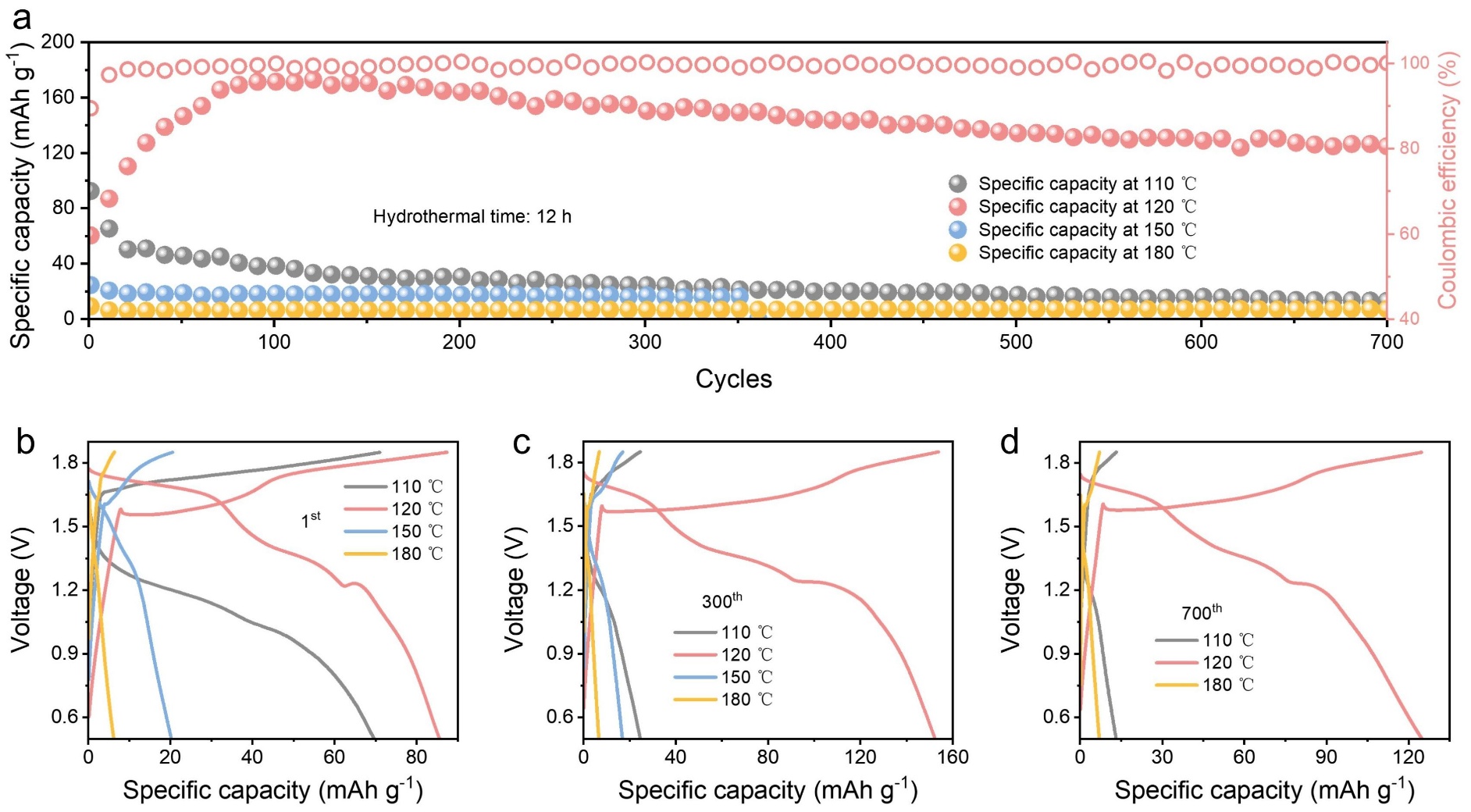


**Figure S16** Electrochemical performance of the Zn-Mn_3_O_4_ batteries at 0.5 A g^-1^ with Mn_3_O_4_ prepared at different hydrothermal temperature. (a) Cycling performance. (b) Charge/discharge curves at the 1^st^ cycle. (c) Charge/discharge curves at the 300^th^ cycle. (d) Charge/discharge curves at the 700^th^ cycle.


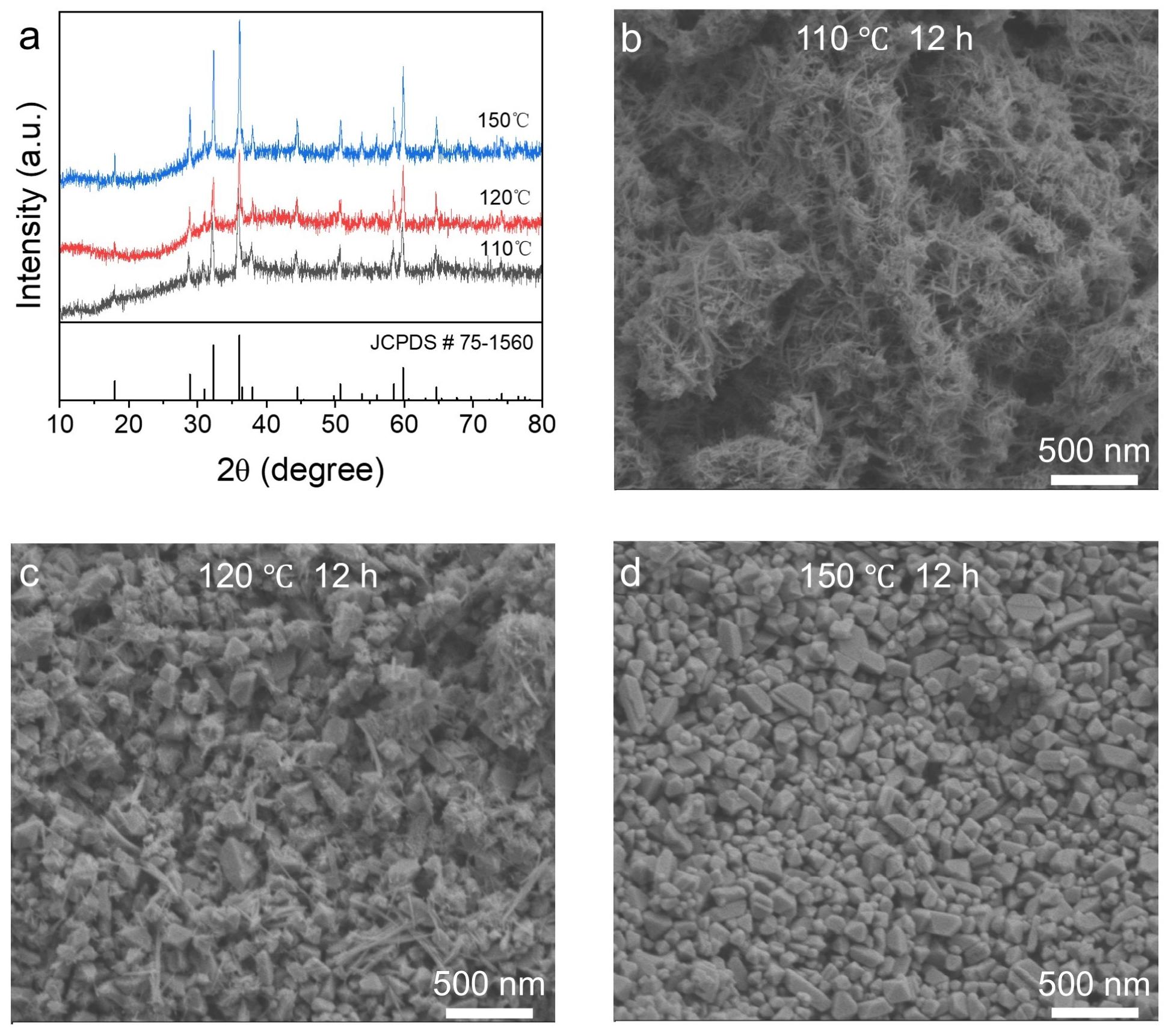


**Figure S17** (a) XRD patterns of the Mn_3_O_4_ material at different hydrothermal temperature. SEM images of the prepared Mn_3_O_4_ at (b) 110 ℃, (c) 120 ℃ and (d) 150 ℃.


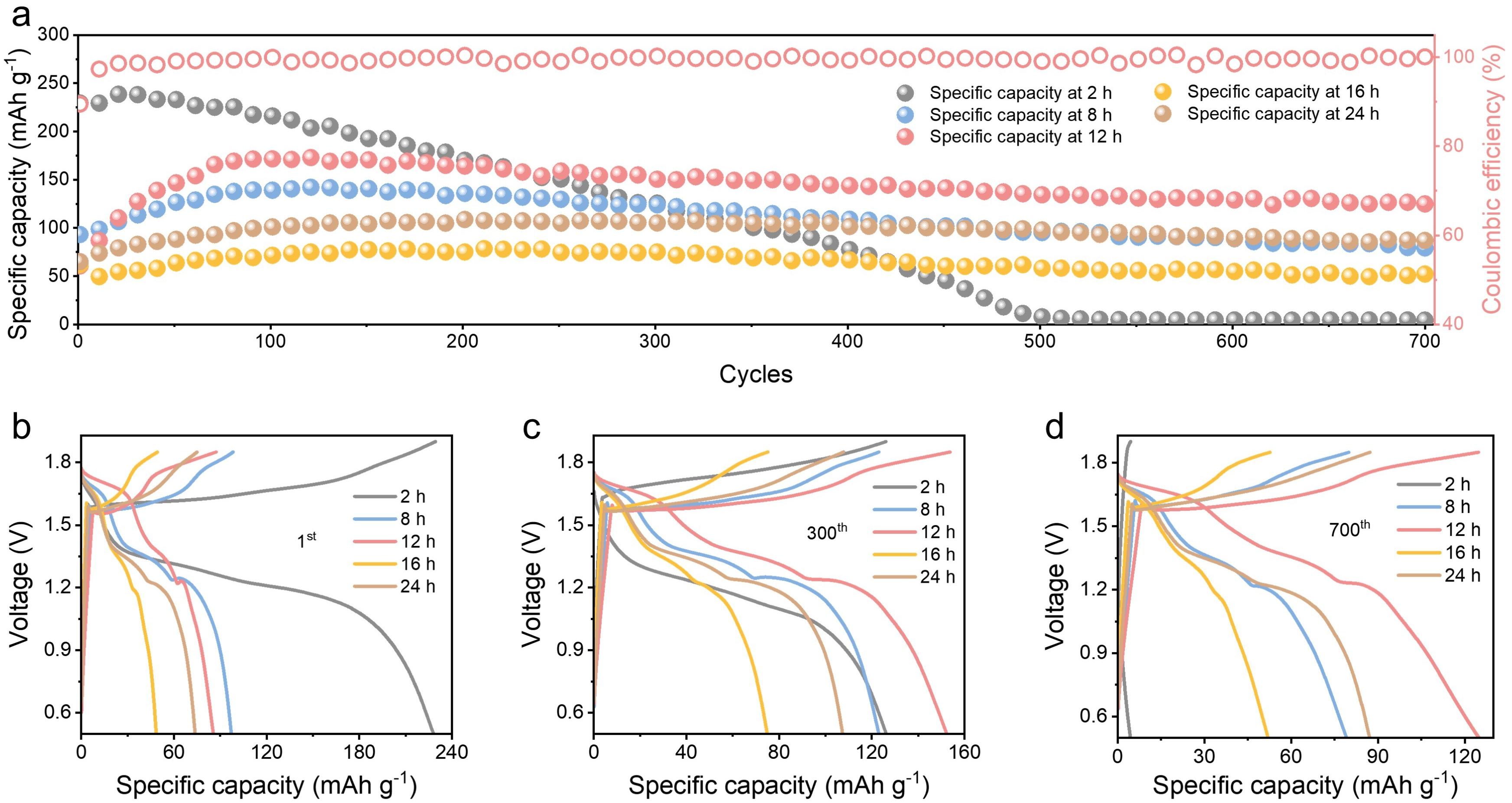


**Figure S18** Electrochemical performance of the Zn-Mn_3_O_4_ batteries at 0.5 A g^-1^ with Mn_3_O_4_ prepared at different hydrothermal time. (a) Cycling performance. (b) Charge/discharge curves at the 1st cycle. (c) Charge/discharge curves at the 300^th^ cycle. (d) Charge/discharge curves at the 700^th^ cycle.


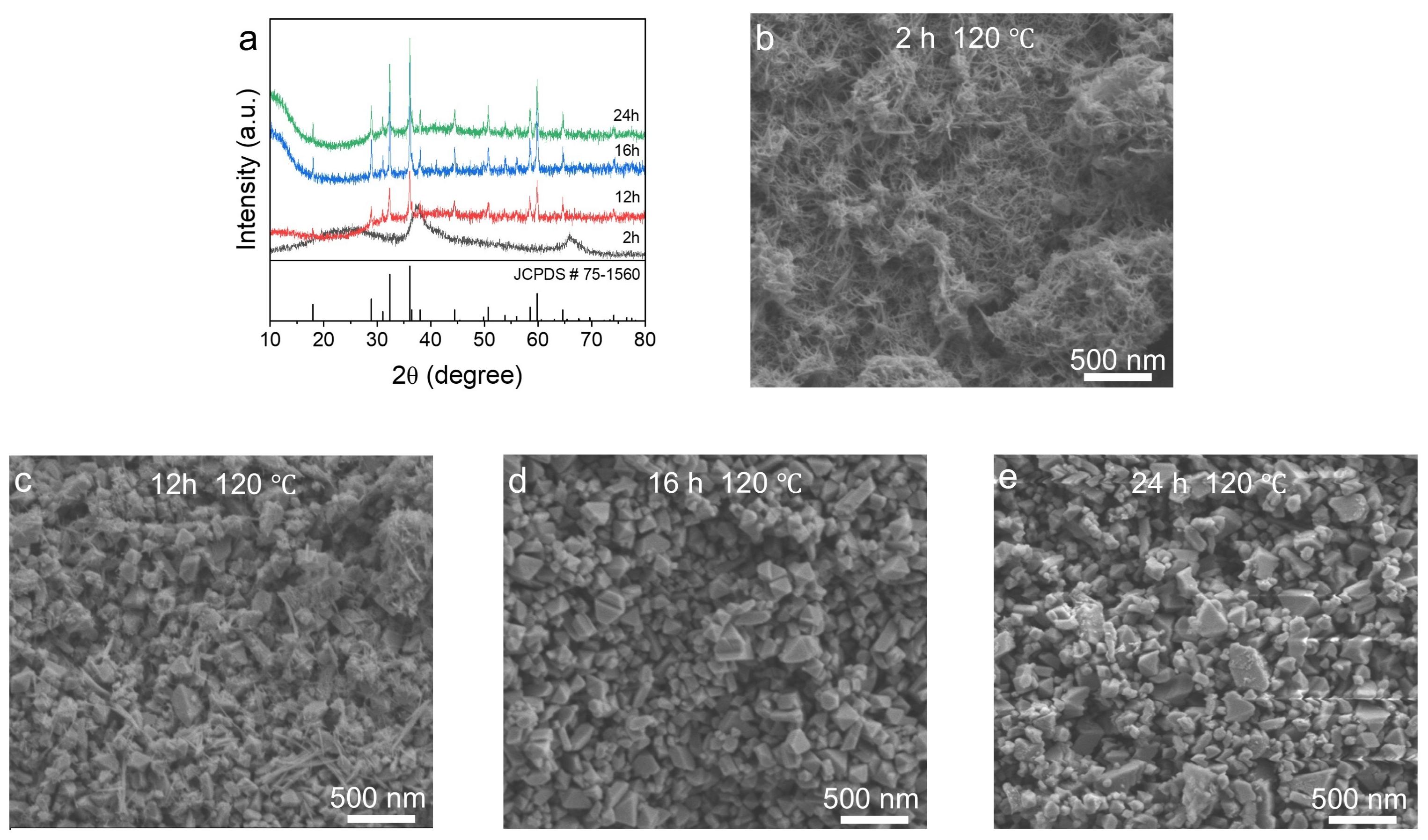


**Figure S19** (a) XRD patterns of the Mn_3_O_4_ material at different hydrothermal time. SEM images of the prepared Mn_3_O_4_ at (b) 2 h, (c) 12 h, (d) 16 h and (e) 24 h.





**Figure S20** Long-term cycling performance of the Zn-Mn_3_O_4_ battery at an areal capacity at 1 mAh cm^-2^.





**Figure S21** Long-term cycling performance of the Zn-Mn_3_O_4_ battery at an areal capacity at 3 mAh cm^-2^.





**Figure S22** Long-term cycling performance of the Zn-Mn_3_O_4_ battery at an areal capacity at 5 mAh cm^-2^.





**Figure S23** Long-term cycling performance of the Zn-Mn_3_O_4_ battery at an areal capacity at 10 mAh cm^-2^.


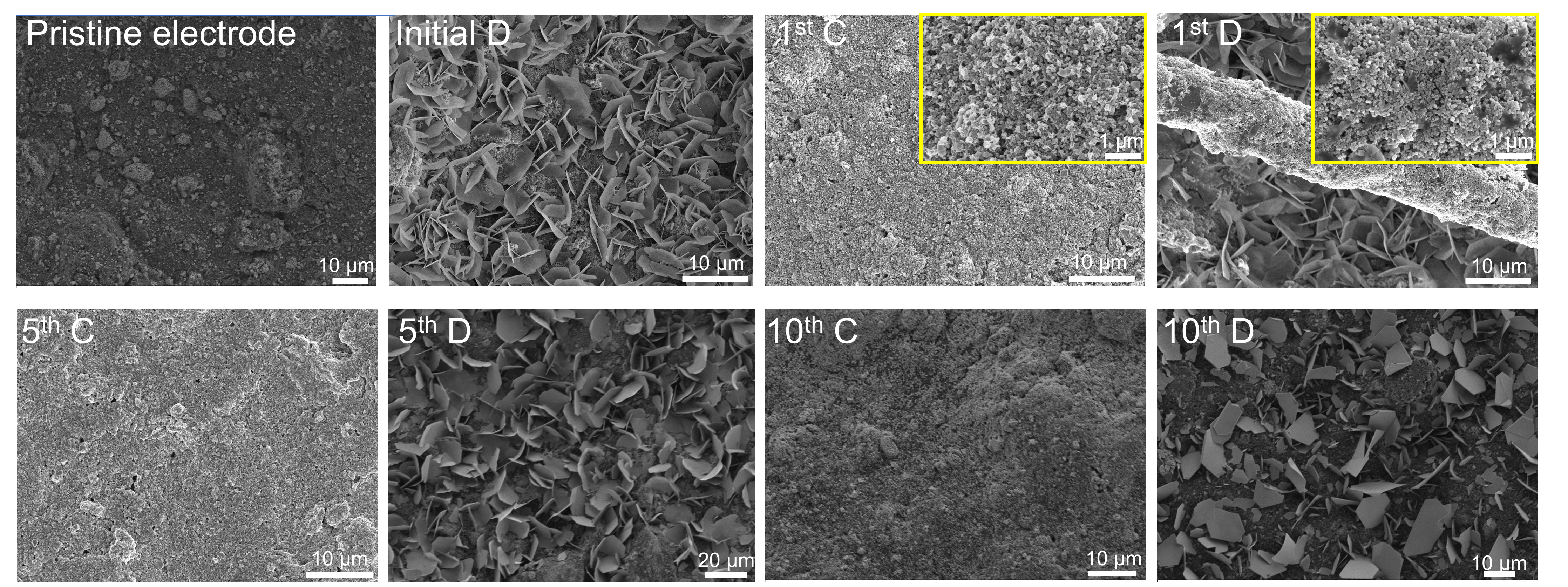


**Figure S24** SEM image images of the Mn_3_O_4_ cathodes at different cycles, where “C” and “D” represent charge and discharge states, respectively.





**Figure S25** Long-term cycling performance of Zn-Mn_3_O_4_ pouch cell with 200 mAh capacity.


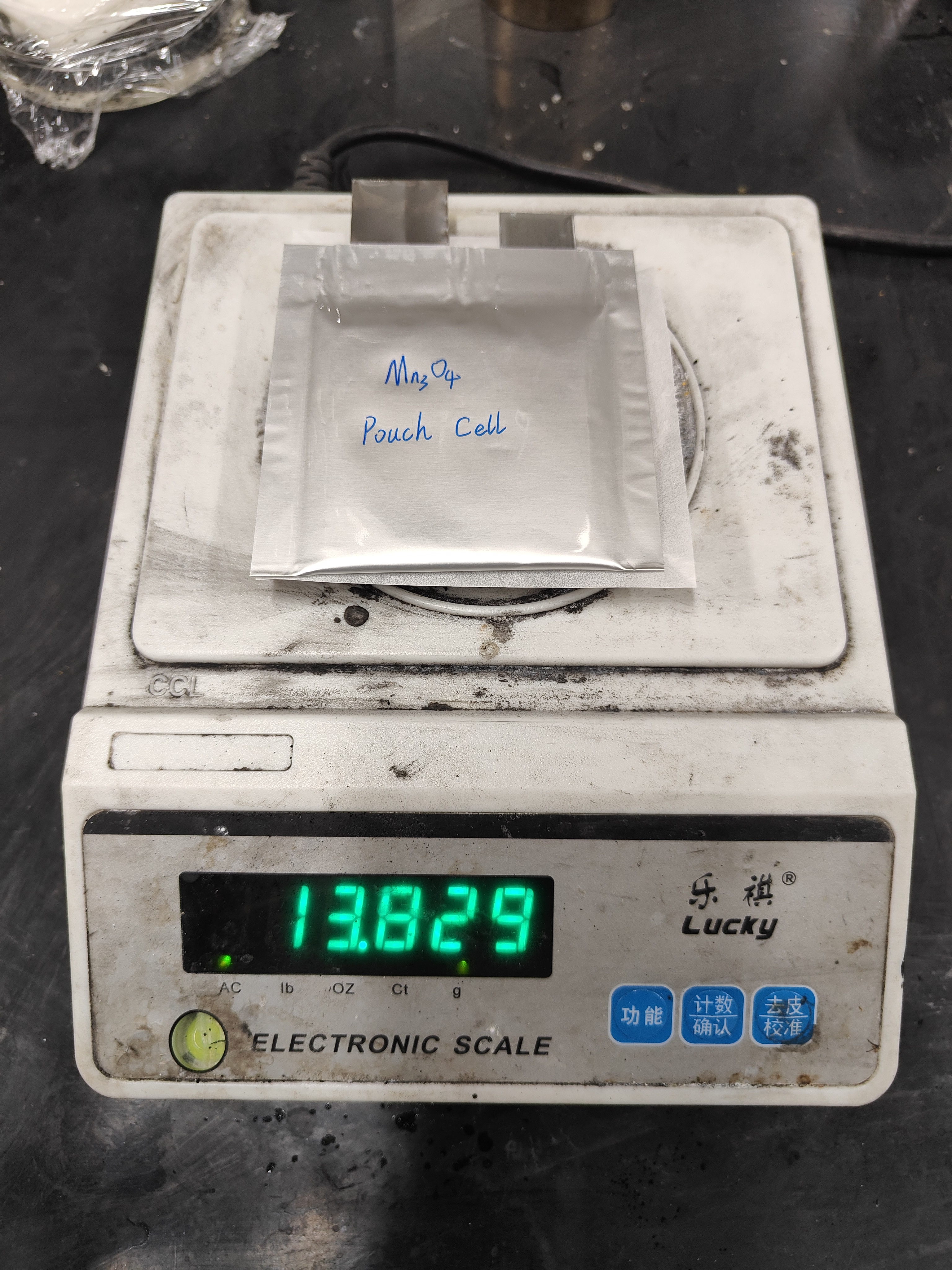


**Figure S26** Digital photo of the weight of the Zn-Mn_3_O_4_ pouch cell measured by an electric microbalance.


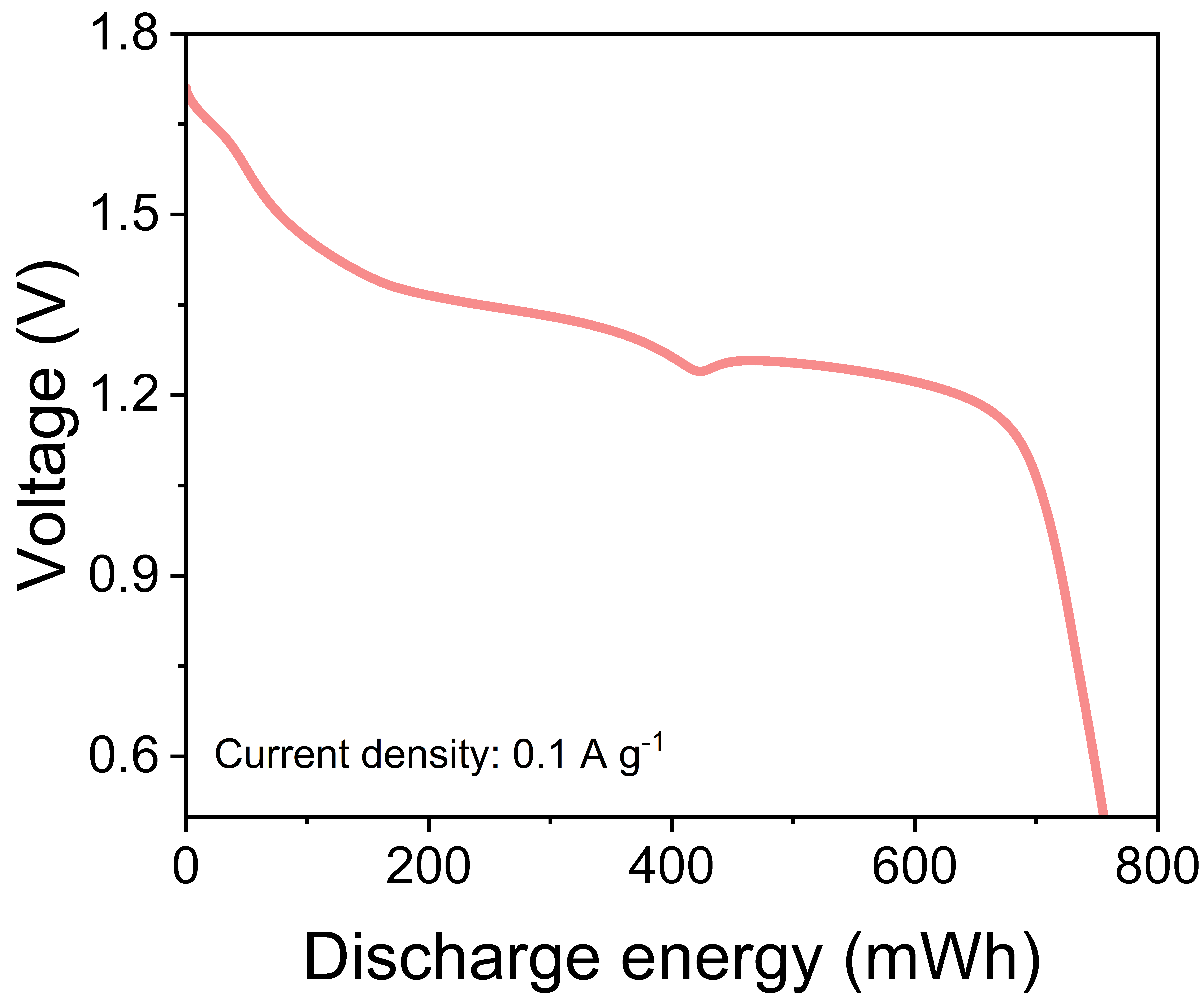


**Figure S27** Discharge curves of the Zn-Mn_3_O_4_ pouch cell for practical energy density calculation.
